# Prevention, treatment and potential mechanism of herbal medicine for Corona viruses: A review

**DOI:** 10.1080/21655979.2022.2036521

**Published:** 2022-02-19

**Authors:** Yan-Xia Liu, Yan-He Zhou, Chang-Hong Jiang, Junyan Liu, Ding-Qiang Chen

**Affiliations:** a Microbiome Medicine Center, Department of Laboratory Medicine, Zhujiang Hospital, Southern Medical University, Guangzhou, Guangdong, China; b Guangzhou Institute of Pediatrics, Guangzhou Women and Children’s Medical Center, Guangzhou Medical University, Guangzhou, 510623 China; c Department of Gastroenterology, Guangzhou Women and Children’s Medical Center, Guangzhou Medical University, Guangzhou, 510623, China; d Department of Civil and Environmental Engineering, University of Maryland, College Park, MD, USA

**Keywords:** Corona viruses, traditional herbal medicine, viral prevention, viral treatment

## Abstract

The pandemic of coronavirus disease 2019 (COVID-19) caused by the SARS-coronavirus 2(SARS-CoV-2) virus has become the greatest global public health crisis in recent years,and the COVID-19 epidemic is still continuing. However, due to the lack of effectivetherapeutic drugs, the treatment of corona viruses is facing huge challenges. In thiscontext, countries with a tradition of using herbal medicine such as China have beenwidely using herbal medicine for prevention and nonspecific treatment of corona virusesand achieved good responses. In this review, we will introduce the application of herbalmedicine in the treatment of corona virus patients in China and other countries, andreview the progress of related molecular mechanisms and antiviral activity ingredients ofherbal medicine, in order to provide a reference for herbal medicine in the treatment ofcorona viruses. We found that herbal medicines are used in the prevention and fightagainst COVID-19 in countries on all continents. In China, herbal medicine has beenreported to relieve some of the clinical symptoms of mild patients and shorten the length of hospital stay. However, as most herbal medicines for the clinical treatment of COVID-19still lack rigorous clinical trials, the clinical and economic value of herbal medicines in theprevention and treatment of COVID-19 has not been fully evaluated. Future work basedon large-scale randomized, double-blind clinical trials to evaluate herbal medicines andtheir active ingredients in the treatment of new COVID-19 will be very meaningful.

## Graphical abstract

**Figure:**
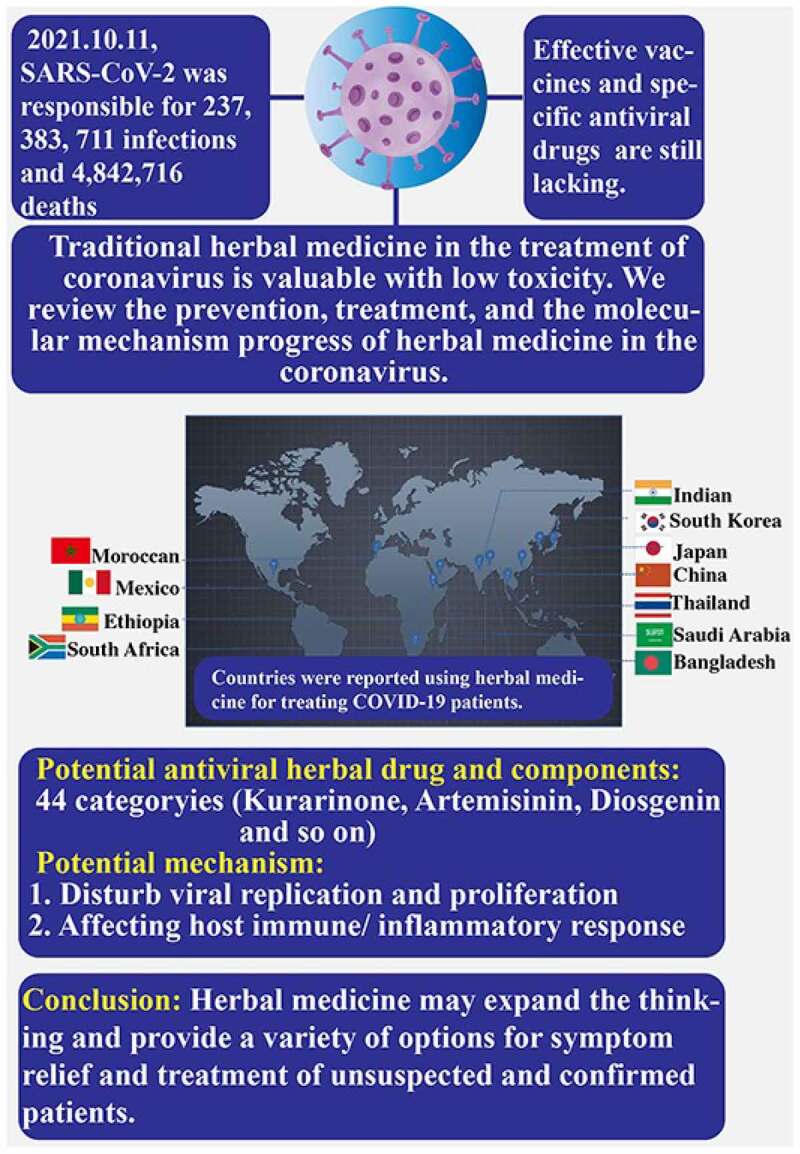

## 1. Introduction

The pandemic of coronavirus disease 2019 (COVID-19) caused by SARS-CoV-2 is an acute respiratory disease with frequent clinical symptoms such as fever, dry cough, and fatigue. Severe patients with high level of cytokines and chemokines can easily present cytokine storm and progress to severe symptoms and death. As of 11 October 2021 COVID-19 has caused 237,383,711 confirmed cases and 4,842,716 deaths worldwide[1]. Obviously COVID-19 has become the greatest global public health crisis since the pandemic influenza outbreak in 1918 [2]. At the same time, the population is generally susceptible to COVID-19, regardless of their age, ethnicity, gender, and health status [3–5], and the protective effect and time of the vaccine still need further investigation [6–8]. The global SARS-CoV-2 infection cases are still increasing. The impact of COVID-19 on global public health challenges and the economic burden of disease continues to worsen [9]. Nevertheless, the leading concern remains that the disease mechanism of COVID-19 has not yet been clarified; effective vaccines and specific antiviral drugs for SARS-CoV-2 are still lacking. Moreover, the specific drugs being developed in various countries cannot pass safety and toxicity tests in a short time, which will further limit the global response to the challenge of COVID-19.

At present, the strategy for the treatment of COVID-19 is still supportive treatment, supplemented by broad-spectrum antibiotics, antiviral drugs, corticosteroids, and plasma from patients with SARS-CoV-2 infection during the recovery period [9]. The conventional drugs such as evusheld, casirivimab, imdevimab, sotrovimab, paxlovid, molnupiravir, lopinavir, ritonavir, chloroquine, and hydroxychloroquine are at different stage of clinical studies, and not as effective as expected. It is worthy of special attention that, in addition to the above-mentioned treatment options, China and South Korea have introduced the use of Chinese medicine in the Chinese herbal medicine COVID-19 treatment guidelines[10]. Chinese medicines are recommended from the third version of Diagnosis and Treatment Guidelines for COVID-19 published by National Health Commission of the People’s Republic of China recommended for the treatment of COVID-19 patients at the stage of disease and symptom differentiation [11]. In the sixth edition of the guide, China recommends Huoxiang Zhengqi (HXZQ) capsules and Jinhua Qinggan (JHQG) granules, Lianhua Qingwen (LHQW) capsules or Shufeng Jiedu (SFJD) capsules for the medical observation period treatment of patients; while patients in the treatment period, follow the issued guidelines and conduct corresponding drug interventions in accordance with the corresponding clinical stages [12]. According to these guidelines, Traditional Chinese Medicine (TCM) treatment covers more than 74,187 (91.5%) confirmed cases in China, most of which were mild and clinical evidence shows that the use of TCM has relieved the symptoms of patients, shortened disease course, and the proportion of patients progressing to severe disease has been decreased [13,14].

The usage of herbal medicines for antiviral treatment can be traced back to ancient Chinese practice cited in Huangdi’s Internal Classic (Huang Di Nei Jing) where preventive effects were recorded. In particular, its application in preventing the spread of SARS caused by SARS-coronavirus (SARS-CoV) and reducing mortality in 2003 has shown great potentials. Inspired by the experience in SARS control, Chinese medicine is widely used during COVID-19 pandemic in China, especially as effective drugs and vaccines are not available. The Guidelines for COVID-19 of China not only recommends that the experience of conventional medicine and TCM in the treatment of SARS and the Middle East Respiratory Syndrome (MERS), but also indicates the treatment of people infected with SARS-CoV-2 is valuable, with effective prevention and treatment for children, adult, and old people infected with SARS-CoV-2 [15–18]. Furthermore, it is reasonable to hypothesize that there will be no adverse reactions [19,20]. Herbal medicine has the characteristics of low toxicity and availability of traditional, making its promising role in the future infectious disease control and treatment [21]. In addition, with the development of computer-based technology in molecule interaction prediction, screening of active compounds targeting viruses or host targets from herbal medicines may be a potential strategy for the treatment of COVID-19 [22,23].

Therefore, in this study, current reports on traditional herbal medicine in the prevention and treatment of COVID-19 were collected and studied, including those recommended by the guidelines, those not included, and the progress in the molecular mechanism of herbal medicine in the treatment of COVID-19. The aim of this study is to gain a simple outline of the application of traditional herbal medicine in the fight against COVID-19, to provide some reference for the use of currently approved antiviral drugs or herbal medicine to treat SARS-CoV-2.

## 2. Usage of herbal medicine against COVID-19 in mainland China

### 2.1 Chinese herbal medicines recommend by Chinese guidelines of COVID-19 treatment

**Figure 1.**
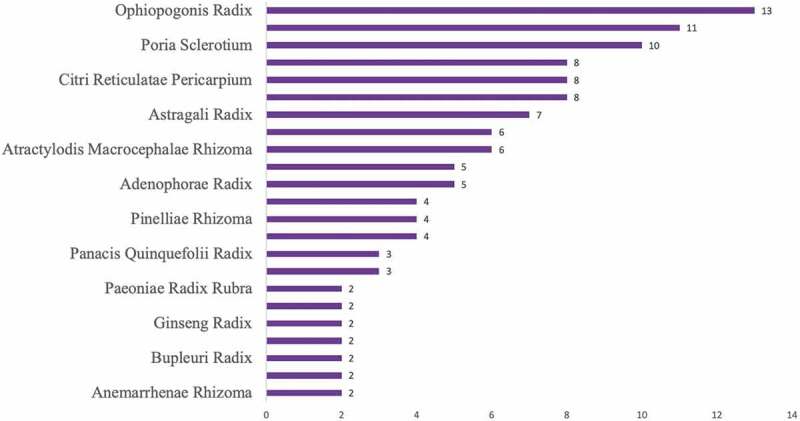
Frequency of commonly use herbs in herbal formulas for treating patients with COVID-19 in recovery stage.

Chinese traditional medicine is a combination of philosophy, ancient disease control and treatment experiences with proved efficacies for different diseases and has developed systematic methods to prevent infectious disease over 2000 years. The overall concept and therapy strategy of Chinese medicine are based on syndrome, physical condition of different people, and the living environmental conditions by means of ‘multi-component, multi-target, multi-pathway’ [24–26]. Chinese medicine believes that COVID-19 is a dampness, blood stasis, and deficiency syndrome, and belongs to the category of ‘epidemic’[27]. In China, COVID-19 patients are recommended to receive different Chinese medicine at different disease stages [27]. Health Commissions of 26 provinces in China have officially recommended the usage of Chinese traditional medicine treatment for COVID-19 patients in combination with conventional medicine, such as Qingfei Paidu Tang (QFPD), *Artemisia annua, Huoxiang, Cangshu, Gancao*, and so on [12]. By summarizing the 8 guidelines on the treatment of COVID-19 with TCM in mainland China, we found that these guidelines recommend a total of 17 herbal formulas with slightly different ingredients. Further analysis of the frequency of these herbs in the guide shows the herbal formulae with the highest frequency of recommendation are the herbal formula of Shen Fu Tang with Su He Xiang Pill or Angong Niuhuang Pill in the severe stage and the combined formula of Xiang Sha Liu Junzi Tang and Li Zhong Pill in the recovery stage. In particular, Angong Niuhuang Pill, Zhi Bao Dan, Zi Xue San, and Su He Xiang Pill are the only prescriptions that were not required in the form of decoction and are only prescribed in the severe stage. On the other hand, the herbal formula of QFPD Tang, which is a combination of 4 different herbal formula with 21 herbs, is recommended by the Chinese national diagnosis and treatment guidelines in the treatment of COVID-19 regardless of disease stage or regional status (Table 1). It is worth noting that among these guidelines, there are only 3 guidelines concerning the treatment of pediatric COVID-19. Probably due to the fact that the total number of infections among children in mainland China. Further analysis of the herbal formulas recommended in these 3 children’s guidelines shows that Yin Qiao San and Xiang Su San are recommended for mild illness; Ma Xing Shi Gan Tang and San Ren Tang can be used for the treatment of moderate illness. Buhuan Jin Zhengqi San, Xuanbai Chengqi Tang and Ganlu Xiaodu Dan are used to treat severe illness. For patients in the recovery period, Liu Junzi Tang + Yu Ping Feng San is also recommended for COVID-19 treatment (Table 1). Table 1 references and integrates the previous literature [28]. We specifically summarized the frequency of TCM findings in these guidelines and found that Ophiopogonis Radix, Poria Sclerotium, and Citri Reticulatae Pericarpium are the most frequently recommended Chinese medicines (Figure 1).

**Table 1.** Summary of Chinese herbal medicines recommended for COVID-19 treatment (Chinese guidelines)

Group	Disease stage	Pattern identification	Name of herbal formula
Adult	Mild	Cold-Dampness Obstructing the Lung	Sang Bei Zhisou San
			Jia Jian Zhengqi San + Da Yuan Yin + Shen Shu San
			Jia Jian Zhengqi San + Huo Po Xia Ling Tang
			Qingwen Baidu Yin + Da Yuan Yin + Huo Po Xia Ling Tang + Sheng Jiang San Jing Fang Baidu San
		Wind-Cold Assailing the Lung	Jing Fang Baidu San + Jiuwei Qianghuo Pill
			Jing Fang Baidu San + Huo Po Xia Ling Tang
		Wind-Heat Invading the Lung	Yin Qiao San + Sheng Jiang San
			Yin Qiao San
			Sang Ju Yin + Yin Qiao San
			Yin Qiao San + Huo Po Xia Ling Tang
		External Cold and Interior Heat	Da Qing Long Tang + Qianjing Weijing Tang
		Warm Pathogen Invading the Lung	Ma Xing Yi Gan Tang + Sheng Jiang San/Da Yuan Yin/Qianghuo Shengshi Tang
			Huo Po Xia Ling Tang + Xiao Chaihu Tang
			San Ren Tang + Sheng Jiang San + Xin Jia Xiang Ru Yin
			Huo Po Xia Ling Tang + Ma Xing Yi Gan Tang
			Sang Ju Yin + Yin Qiao San
			Xiao Chaihu Tang + Sang Xin Tang + Jiuwei Qianghuo Tang + Huoxiang Zhengqi San
			Ganlu Xiaodu Dan + Huoxiang Zhengqi San
			Ma Xing Yi Gan Tanga + Da Yuan Yin
			Ma Xing Yi Gan Tang + Sheng Jiang San + Da Yuan Yin
			San Ren Tang + Huo Po Xia Ling Tang
			Ma Xing Yi Gan Tang + Sheng Jiang San + Da Yuan Yin
		Heat Pathogen Assailing the Lung	Da Yuan Yin
			Ma Xing Shi Gan Tang + Da Yuan Yin
			Yin Qiao San + Qingwen Baidu Yin
			Yin Qiao San + Ma Xing Shi Gan Tang
			Ma Xing Shi Gan Tang + Xiao Chaihu Tang
		Dampness Encumbering the Exterior and Interior	Ma Xing Yi Gan Tang + Da Yuan Yin + Sheng Jiang San + Xiang Ru Yin
			Huo Po Xia Ling Tanga + Yin Qiao San
			Ma Xing Yi Gan Tanga + Huo Po Xia Ling Tang
		Pathogen Invading the Stomach and Intestines	Wang Shi Lian Po Yin + Huo Po Xia Ling Tang
		No specific PI	Qingfei Paidu Tang
	Moderate	Warm Heat Pathogen Congesting the Lung	Ma Xing Shi Gan Tang
			Ma Xing Shi Gan Tang + Yin Qiao San
			Ma Xing Shi Gan Tanga + Ganlu Xiaodu Dan + Sangbaipi Yin
			Ma Xing Shi Gan Tang + Qianjing Weijing Tang
			Qingqi Huatan Tang + Huo Po Xia Ling Tang
			Ma Xing Shi Gan Tang + Ganlu Xiaodu Dan + Sheng Jiang San
		Exterior Cold and Interior Heat Complicated by Dampness	Ma Xing Shi Gan Tang + Chuwen Huadu Tang + Sheng Jiang San
			Chaihu Da Yuan Yin + Leng Xiao Pill
		Dryness Invading the Lung	Sang Xing Tang + Qingzao Jiufei Tang
		Cold-Dampness Obstructing the Lung	Ma Xing Yi Gan Tang
			Jia Jian Zhengqi Sana + Da Yuan Yin
		Dampness Obstructing Middle Energizer	Qianjing Weijing Tang + San Zi Yangqin Tang + Shengyang Yiwei Tang
			Yi Jia Jian Zhengqi San
		Dampness Obstructing the Lung	Ma Xing Shi Gan Tanga + Qingqi Huatan Tang + Sangbaipi Yin
			Ma Xing Yi Gan Tang + Da Yuan Yin
			Ma Xing Yi Gan Tang + Sheng Jiang San + Da Yuan Yin
		Dampness toxin Congesting the Lung	Ma Xing Yi Gan Tanga + Da Yuan Yin
		Phlegm-Heat Congesting the Lung	Qingwen Baidu Yin + Sheng Jiang San
			Ma Xing Shi Gan Tang + Ganlu Xiaodu Dan + Sheng Jiang San
			Xuan Bai Cheng Qi Tang + Huanglian Jiedu Tang + Jiedu Huoxue Tang
			Ma Xing Yi Gan Tang + Sheng Jiang San + Da Yuan Yin
			Ma Xing Shi Gan Tang + Qingqi Huatan Tang
		Epidemic Toxin Blocking the Lung	Ma Xing Gan Shi Tang Da Yuan Yin + Sheng Jiang San + Xuan Bai Cheng Qi Tang
			Xuan Bai Cheng Qi Tang + San Xiao Yin + Huo Po Xia Ling Tang
			Ma Xing Gan Shi Tang + Da Yuan Yin
			Ma Xing Shi Gan Tang + Huo Po Xia Ling Tang + Sheng Jiang
			San + Huanglian Wendan Tang
			Xuan Bai Cheng Qi Tang + Da Yuan Yin
		Heat Pathogen Assailing the Lung	Xuan Bai Cheng Qi Tang + Huanglian Jiedu Tang
			Ma Xing Shi Gan Tang
			Bai Hu Tang + Qingying Tang + Qingwen Baidu Yin
			Ma Xing Shi Gan Tang + Yin Qiao San
		No specific PI	Qingfei Paidu Tang
	Severe	Internal Block and External Collapse	Shen Fu Tang + Su He Xiang Pill/Angong Niuhuang Pill
			Shen Fu Tanga + Su He Xiang Pill/Angong Niuhuang Pill/Zi Xue San
			Shen Fu Tang + Su He Xiang Pill/Angong Niuhuang Pill/Shexiang Niuhuang Pill
			Shen Fu Tang
			Sheng Mai San + San Shi Tang + Angong Niuhuang Pill
			Si Ni Jia Renshen Tang + Angong Niuhuang Pill/Zi Xue San
			Si Ni Jia Renshen Tang + Zhi Xue San/Su He Xiang Pill/Angong Niuhuang Pill
			Xuanbai Chengqi Tang
		Toxin Blocking the Lung	Ma Xing Shi Gan Tang + Xuanbai Chengqi Tang
			Ma Xing Shi Gan Tang + Sang Bei San
			Xuan Bai Cheng Qi Tang + Sang Bei San
			(Self-prescribed) Tingli Xiefei Tang
			Shen Fu Tang
			Shen Fu Tang + Su He Xiang Pill/Angong Niuhuang Pill
			Xuanbai Chengqi Tang + Huanglian Jiedu Tang + Jiedu Huoxue Tang
			Qingwen Baidu Yin + Jiedu Huoxue Tang
			Huashi Baidu Formula
		Blazing of both qi and nutrient	Qingwen Baidu Yin
		Toxin Flowing through Five Viscera	Xiang Sha Liu Junzi Tang + Shen Su Jiangqi Tang
		Depletion of Essential Qi	Poge Jiuxin Tang + Gualou Xiebai Banxia Tang + Danshen Yin
		Exhaustion of Yin and Collapse of Yang	Shen Fu Long Mu Tang
		Toxin Clouding the Orifices	Zi Xue Dan/Zhi Bao Dan/Angong Niuhuang Pill/Su He Xiang Pill
		Cold Epidemic Blocking the Lung	Xiao Qinglong Tang
		No specific PI	Qingfei Paidu Tang
Children	Mild	Seasonal Epidemic Invading the Exterior-defense	Yin Qiao San
			Xiang Su San
	Moderate	Dampness-Heat Blocking the Lung	Ma Xing Shi Gan Tang+ San Ren Tang
	Severe	Dampness-Heat in the Spleen and Stomach	Buhuan Jin Zhengqi San
		Heat Toxin Blocking the Lung	Xuanbai Chengqi Tang + Ganlu Xiaodu Dan

**Figure 2.**
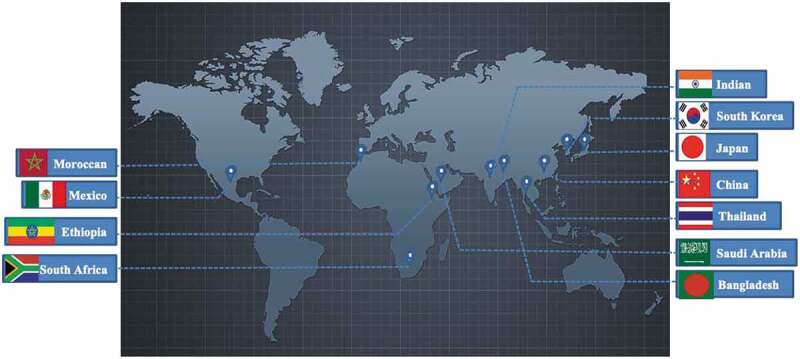
Countries were reported using herbal medicine for treating COVID-19 patients.

### 2.2 Officially issued Chinese medicine prevention recommendations for COVID-19

Treating the pre-diseased condition has been considered as one of the most important principles in Chinese traditional medicine [29]. The main principles of Chinese medicine were to prevent external pathogens, disperse winsd, dissipate heat, and relieve dampness. Historically, Chinese medicine approaches, for example oral administration of preventive herbal formulae, were recommended for infectious disease controlling [30]. As early as the 1970s, the study of Chinese traditional medicine against the influenza virus has been investigated in China [31,32]. As for COVID-19, till date there is no specific medicine largely recommended by WHO regarding COVID-19 treatment. Twenty-three provinces in China mainland have issued Chinese medicine programs, among which are mainly oral formula. COVID-19 prevention prescriptions based on the theory of ‘Natural Factor’ were used to maintain vital ‘Qi’. Modern pharmacological studies have shown that TCM ingredients are effective in clearing away heat and detoxifying, as well as in relieving surface dampness, thereby inhibiting the virus. The most frequently used herbs included Radix astragali (*Huangqi)*, Radix glycyrrhizae (*Gancao*), Radix saposhnikoviae (*Fangfeng)*, Rhizoma Atractylodis Macrocephalae (*Baizhu*), Lonicerae Japonicae Flos (*Jinyinhua*), and Fructus forsythia (*Lianqiao*) [31]. Among these ingredients, *Huangqi*, which is well known for its protective effects on ‘Qi’, together with *Fangfeng* and *Baizhu*, are all ingredients of a classical herbal formula Yupingfeng Powder [29]. Several studies have mentioned their role in SARS prevention and syndrome improvement. Some studies supposed that Yupingfeng Powder may have antiviral, anti-inflammatory and immunoregulatory effects [29]. The ingredient glycyrrhizic in *Gancao* has been suggested to prevent SARS infection [33]. *Jinyinhua* and *Lianqiao* are the core of components of Yinqiao Powder, which was considered to be a formula used to prevent and treat respiratory infectious diseases in ancient [34]. These studies provide evidence of Chinese herbal medicine for COVID19 prevention. So far, China has issued a total of 7 editions of the guidelines, in the sixth edition of the guide, HXZQ capsule and JHQG granules, LHQW capsule or SFJD capsule are recommend for the medical observation period treatment of patients [12]. In the absence of specific treatment drugs for COVID-19, most medical professionals in China have a positive attitude toward TCM [35].

## 3. Usage of herbal medicines against COVID-19 outside China Figure 2

### 3.1 Asia

#### East Asia

In South Korea, the Association of Korean Medicine and the Korean Association of Traditional Pulmonary Medicine each issued the first version of traditional medicine guidelines on the prevention and treatment of COVID-19 at the end of February 2020 [36]. The Korean guidelines suggested 4 PIs and 15 herbal formulae for the mild stage, 3 PIs and 3 herbal formulae for the severe stage, and 2 PIs and 2 herbal formulae for the recovery stage [37]. Herbal medicines such as Qing-Fei-Pai-Du-Tang, Yin-Qiao-San and Huo-Xiang-ZhengqiSan were prescribed by Korean Medicine doctors according to established protocols made by expert advisory panels and by foreign guidance or data on COVID-19. 30% herbal medicines prescribed through telemedicine center were Qing-Fei-Pai-Du-tang [38]. South Korea recommends the use of Youngyopaedoc San plus Bojungikgi Tang and Youngyopaedoc San plus Saengmaek San for patients with a history of SARS-CoV2 exposure based on its own national conditions; Youngyopaedoc San plus Bulhwangeumjeonggi San, and Youngyopaedoc San plus Bojungikgi Tang were recommended for treatment of mildly symptomatic COVID-19 patients; For patients without pneumonia but with wind-warmth disease invading the lungs, Youngyopaedo San plus Galgunhaegui Tang was recommended; While for those with dampness-heat disease in the lungs, Sosiho-tang plus Bulhwangeumjeonggi San was recommended. Samchulkunbi Tang plus Saengmaek San, or Samchulkunbi Tang plus Chungseuiki Tang was recommended for the recovery stage [37]. In Japan, there are attempts to use Matoto to treat COVID-19. And the Japanese traditional (Kampo) medicine, kakkontokasenkyushin’I was used and could improve the numeric rating scale scores of the smell impairment [39,40]. Another Japanese Kampo medicine ninjin’yoeito (NYT), has been showed effective in treating patients with severe COVID-19 in ICU [41]. It is noted that the Health Ministry of Thailand approved *Andrographis paniculata* extract for COVID-19 treatment, which increase adapt- ability, the state of nonspecific resistance, resilience, and survival of organisms. And the combination of *Andrographis paniculata* with Maoto, Maoto-ka-senshinren, seems most promising for the treatment of viral pandemics [42].(Figure 2)

#### Saudi Arabia

Based on the recognition that herbal medicines are generally safe, herbal medicines are commonly used in the Far East, Near East and the Middle East to improve immunity and prevent viral infections. The Saudi public has a high rate of use of herbal medicines and dietary supplements [43,44]. A small study showed that more than half of the people participating in the survey, mainly Saudis, used herbs (mainly garlic and cinnamon) and/or dietary supplements to strengthen the immune system to prevent COVID-19 infection during the COVID-19 period [43,44]. In addition, there are also herbs such as *Nigella sativa oil* (NSO) for COVID-19 treatment in the clinic [45]. The result of an open-label randomized controlled clinical trial revealed that patients with mild COVID-19 infection *Nigella sativa* get faster recovery of symptoms than usual care alone. *Nigella sativa* is a kind of ‘miracle herb’ which is very popular in various traditional systems around the world [46]. It contains a number of bioactive components, of which, thymoquinone has been proven to have anti-viral activity, and Zn salt supplement might enhance its effects [47]. Molecular docking result showed that *Nigellide* and α *hederin*, the main compounds of *Nigella sativa*, can dock with Mpro and might serve as potential inhibitors of SAR-COV-2 [48]. Its bioactive components such as thymoquinone which was proven to have anti-viral activity. Although detail mechanism is still limit, current findings suggest the potential therapeutic benefits in anti-COVID-19, moreover, encourage more clinic attempt of herbal medicine usage and and mechanism study of *Nigella sativa* in the future.

#### Bangladesh

Based on its own medical resources, Bangladesh regards the prevention of the spread of the virus as its main strategy to fight COVID-19. Measures include isolation at home, wearing masks, maintaining social distancing, and washing hands regularly. At the same time, it is recommended to take vitamins and herbs for prevention. In the herbal medicine section, most people choose to drink green tea and black tea. Others also include herbal foods such as ginger, black seeds, honey and cloves alone or mixed with tea or hot water, although their antiviral activity needs to be further demonstrated [49].

#### India

Ayurvedic medical system and Siddha system of medicine are important parts of Indian medical system. The Ayurvedic medical system treats the body as a whole rather than the symptoms of the disease, so it may play a very important role in the fight against SARS-CoV-2. For example, the herbal compounds in Ayurveda such as silymarin, palmatine, curcumin, and syringin have potential antiviral activity and may play a potential role in COVID-19 pandemic [50–52]. In vivo zebrafish model based on the SARS-CoV-2 infection also showed the potential antiviral effect of calcio-herbal medicine Divya-Swasari-Vati, which is used to treat chronic coughs and common colds in Indian [53]. Siddha system of medicine is a system that focuses on solving the root cause of the disease rather than treating the symptoms of the disease, and advocates the use of herbal, mineral and animal-derived drugs to treat the disease. Kabasura Kudineer, which consists of 15 herbal ingredients is indicated for use in Aiya suram (fever) and Aiya noigal (respiratory diseases) and recent clinical evidence showed that it reduced viral load in asymptomatic patients [54].

### 3.2 Africa

Due to differences of the level of social and economic development and health care infrastructure in various regions, not all countries can adopt current medical technologies and obtain sufficient vaccines and therapeutic drugs. Therefore, in some regions, such as Africa, herbal medicine plays an important role in the fight against COVID-19 [55]. As a traditional medicine continent with long history, Garlic, ginger, lemon, garden cress and ‘Damakase’ were frequently used for COVID-19 treatment in Africa. Seeds and leaves were mainly from home gardens, and were frequently used parts. However, the efficacy and safety might be looked into by pharmaceutical industries and researchers.

In South Africa, Artemisia afra tea without artemisinin 1 is widely used [56]. In Ethiopia, common herbs such as garlic, ginger, lemon, cress and damakase are also used to treat COVID-19 [57].

#### Morocco

Morocco is a country in the Mediterranean region, with a diverse climate and rich traditional experience of medicinal plants [58]. Research shows that there are 20 plant species had been most frequently used for the prevention and treatment of COVID 19 in Salé Prefecture. The most commonly used are *Eucalyptus globulus Labill., Azadirachta indica A. Juss*., and *Ziziphus lotus (L.) Lam* [59,60].

### 3.3 America

Mexico is the country with the fourth largest biodiversity in the world and the country with the largest number of medicinal plants after China. Mexican ethnomedicine also has a deep-rooted tradition. Its research on phytochemistry and pharmacology has been over 100 years, and it is the branch of TCM in herbal medicine research [61]. Nadia et al. analyzed the potential binding of 100 compounds isolated from Mexican herbs to the SARS-CoV-2 virus, and found that emodin anthrone, kaempferol, quercetin, esculin, cichorin, luteolin, matrix. These 10 ingredients have potential antiviral function, although the safety and effectiveness need to be further verified [62]. It is necessary to study the effect, influence, and the mechanism of the ingredients with potential antiviral function.

## 4. SARS-CoV-2 infection

### 4.1 SARS-CoV-2 Life cycle

Coronavirus are enveloped, single-stranded RNA virus. They are common human pathogen. Coronavirus has no segments and is the largest RNA virus known to date (genome size is 26 to 32 kilobases), consists of a nucleocapsid (composed of genomic RNA (gRNA) and phosphorylated nucleocapsid protein (N)) and outer envelope proteins (including membrane (M) protein, envelope (E) protein and spike (S) protein). Since viruses have the characteristics of a corona appearance, they are named as coronaviruses. In terms of genotype and serology, coronaviruses are divided into four genera: α, β, ɣ and δ coronavirus. Among them, β-coronavirus is divided into four virus lineages, A, B, C and D. Only α- and β-type coronaviruses can cause human infections. For example, SARS-CoV that broke out in 2003 and Middle East Respiratory Syndrome coronavirus (MERS-CoV) that broke out in 2013 belong to the β-coronavirus family.

SARS-CoV-2, which causes COVID-19, belongs to β-coronavirus with a genome length of about 30 kb and with a 5’-cap structure and a 3ʹpoly(A) tail [63,64]. It has 80% and 50% homology with SARS-CoV and MERS-CoV [65,66]. Virus infection is initiated by the binding of the S protein to the host angiotensin-converting enzyme 2 (ACE2) receptor. Subsequently, the S protein is processed into S1 and S2 subunits, which are responsible for cell invasion by the cellular serine proteases TMPRSS2 [67]. When the virus uses S protein bind to ACE2 to invade respiratory epithelial cells by endocytosis, on the one hand, SARS-CoV-2 gRNA translated pp1a and pp1ab polypeptides and processed to produce 16 non-structural proteins (nsps) by chymotrypsin-like cysteine protease (3CL-PRO/Mpro) and the N-terminal end is processed by the papain-like protease (PLpro). Nsps are important for viral propagation. Of the 16 nsps, 12 nsps harboring the RNA-dependent RNA polymerase (Rdrp) is an essential enzyme for viral replication. On the other hand, viral gRNA can also be used as a template for replication and transcription to form progeny gRNA and encode structural proteins S protein, E protein E, M protein, and N protein and accessory proteins 1ab, 3a, 6, 7a, 7b, 8, and 10 and other sub-gRNAs. The newly produced gRNA is further packaged into virus particles and released outside the cell, continues to infect other cells or spread to other healthy people, entering the next replication cycle [63,68].

### 4.2 The effect of SARS-CoV-2 infection on human being

Once the infection is confirmed by the virus, in clinical manifestations, the common symptoms of SARS-CoV-2 infection are fever, cough and myalgia or fatigue; occasionally, sputum production, headache, hemoptysis and diarrhea. On admission, laboratory examination showed a decrease in the number of lymphocytes and ground-glass opacity was the most common radiologic finding on chest computed tomography (CT). A clinical analysis of patients from Wuhan, Mainland China showed that the median time from onset to dyspnea was 8 days [69]. SARS-CoV-2 invades the respiratory system to establish infection and can cause pneumonia. Although most infected patients have mild clinical symptoms, further development can be complicated by acute respiratory distress syndrome, acute heart injury and secondary infection. In severe cases, it can progress to severe illness or even death [69]. At the molecular level, viruses employ host cell’s recourse and translation machinery for the production of their proteins and infectious progeny to finish their life cycle. Moreover, host protein synthesis is also critical for the host cell’s immune response to virus infection by innate immune system. Hence, it is not surprising why several positive RNA viruses regulate host protein synthesis so as to inhibit the translation of cellular mRNAs and benefit the production of viral proteins [70]. Indeed, expression of several coronavirus proteins, including the heavily glycosylated S protein, was shown to induce ER stress, which was also observed in coronavirus-infected cells. S proteins of both SARS-CoV and IBV were found to physically interact with eIF3F, to modulate host translation, including the expression of the pro-inflammatory cytokines interleukin (IL) 6 and 8, at a later stage of infection [71]. Importantly, the nsp1 proteins of both α- and β-coronaviruses were identified as inhibitors of multiple steps of translation initiation [72,73]. The latest evidence shows that the nsp1 proteins of SARS-CoV-2 can bind to the ribosomal RNA of human Hela cells to inhibit viral genome translation [74]. On the other hand, the replication of coronavirus is regulated by a variety of host factors, which induce changes in cell structure and cell signaling pathways. The abnormal expression of key genes and the activation of key signal pathways during HCoV infection can help induce antiviral immune response and cause coronavirus-related symptoms. For example, SARS-CoV infection leads to reduce of p53 expression levels. Interestingly, in cells lacking p53 expression, the replication level of SARS-CoV was significantly increased [75]. Recent studies on the host factors associated with SARS-CoV-2 infection have found that the use of CRISPR technology to screen the alarm protein HMGB1 and SWI/SNF chromatin that are related to the promotion of virus replication and infection functions reshape the TGF-β signaling pathway [76]. In severe cases of SARS-CoV the pathology was associated with aberrant or hyper-activation of innate immune signaling. This resulted in the aberrant production of interferons and high levels of pro-inflammatory cytokines such as IL-1, IL-6, IL-8, CXCL-10 and TNF-alpha in the lungs [77]. After SARS-Cov-2 infection, activation of pro-inflammatory cytokines TNF-α, IL-6, CCL-2/MCP-1 and CXCL-10/IP-10 was also found [69,78]. Some of these cytokines mediate different cell signaling pathways through mediated by JAKs, PI3K-AKT, MAPK, and NF-κB pathways. In addition, recently evidence suggested that SARS-CoV-2 infection can cause apoptosis and cell survival related Akt/mTOR/activation in an in vitro infection model while HIF-1 decreases [79]. It is possible to block autophagy and apoptosis to overcome infection-related pressure to maintain protein synthesis [80].

## 5. Potential anti-COVID-19 pharmacological components

Due to the lack of specific drugs for COVID-19, herbal/plant bioactive derivative molecules have become an important alternative to the treatment of COVID-19. Kurarinone is an amyl flavonoid, used as an analgesic in traditional Asian medicine, isolated from the root of the Asian shrub Sophora flavescens. It was recently discovered that Kurarinone can induce autophagy to inhibit coronavirus infection. And it can also inhibit the virus-induced cytopathic effect, extracellular and intracellular viral RNA and viral protein expression. At the initial infection stage, kurarinone may be considered to be a novel and useful prophylaxis for the treatment of coronavirus infection [81].

In the treatment of kidney injury in Chinese herbal medicine related to COVID-19, *Astragalus, Poria, White Bamboo, Rehmannia Glutinos*a, and *Chinese Yam* are the core medicinal materials, which might regulate AGE-RAGE, PI3K- Key proteins of AKT, TNF, apoptosis and other pathways [82]. Herbal extracts such as *Anupana* containing epicatechin and hesperidin may have potential antiviral effects [82]. Molecules from kalmegh provide immune protection and antiviral responses by involving different pathways, such as the Toll-like receptor pathway, the PI3/AKT pathway, and the MAP kinase pathway for COVID-19 infection [52]. In addition, simulation experiments also found that Moupinamide in Piper nigrum, a component of Ayurvedic decoction, can strongly bind to the Spik protein of SARS-Cov-2 [83]. The purified extract of curcumin and piperine is a patented natural compound, which is a combination of black pepper extract and curcumin extract. In addition to piperine and curcumin, the formula also contains a variety of active ingredients, including pentaerythritol, sitosterol, termerone, lupeol, amygdalin and vitamin D3 with potential immunomodulatory and antiviral effects of SARS-CoV-2 [84]. Puerarin and quercetin can significantly affect the binding of the virus S protein to the ACE2 receptor. It is worth noting that quercetin can also bind to the RBD domain of the S protein, which indicates that quercetin not only has a receptor blocking effect, but also has a virus-neutralizing effect on SARS-CoV-2 [85]. Nigella sativa, belonging to family Ranunculaceae, is known as a curative herb for all ailments except death, its nucleocapsid protein NRBD and SARS-CoV-2 PL-PRO have been revealed as important drug targets for Nigella sativa plant ingredients. The most effective plant ingredients namely. Campesterol, eucalyptol, α-spinachsterol and β-sitosterol show high affinity for SARS-CoV-2ʹs NRBD and PL-PRO. Due to their bioavailability, drug similarity and almost zero toxicity and mutagenicity, these plant components can be further explored as potential antiviral agents for the treatment of COVID-19 in vitro and in vivo [86]. Based on network pharmacology analysis, combined with clinical treatment observations, Du et al. found that Epigallocatechin-3-gallate, the active ingredient of the three TCMs Yangyin Jiedu (YYJD), Dayuan Xiaodu (DYXD) and Chaihu Qingzao (CHQZ), targets 3CLpro, has the potential to interfere with SARS-CoV-2 The role of life cycle [87].The analysis of plants commonly used in Mexico such as cactus, Nadia et al. showed that quercetin, riolozatrione and cichorium can target the key protein of SARS-CoV-2. Among them, cichorium is safer and bind to Rdrp, Nsp14, Nsp3. Moreover, these compounds were predicted to reach acceptable concentrations in both peripheral blood and cells [62]. Ephedrine, pseudoephedrine and methylephedrine are the active ingredients of a variety of TCMs, which can bind to some amino acid residues with ACE2 to inhibit the pseudovirus in the SARS-CoV-2 pseudovirus model Enter HEK293T cells overexpressing ACE [88]. Coronil is an herbal extract that contains Solanum lycopersicum, Penicillium heart and Tamarind, with potential antiviral effects. Laboratory evidence shows that Coronil prevents SARS-CoV-2 S protein-mediated virus from entering A549 cells by inhibiting the spike protein-ACE-2 interaction, and significantly reduces the levels of IL-6, IL-1β and TNF-αlevel [88]. Researchers analyzed the secondary metabolites of Brazilian herbs and showed that triterpenoids and phenolic compounds can bind to SARS-CoV-2 and have potential antiviral functions [89]. Monika et al. predicted that Fagaronine, Isoboldine, Sageone, Lycorine and Wogonin in plants combined with Mpro [90]. The herbal infusion horchata from Ecuador has more than 50 compounds. Among them, benzoic acid, 2-(ethylthio)-ethyl ester, l-Leucine-N-isobutoxycarbonyl-N-methyl-heptyl and isorhamnetin are candidate compounds for Mpro inhibition, and isorhamnetin-3-O-(6-Orhamnosyl-galactoside), dihydroxy-methoxyflavanone and dihydroxyphenyl)-5-hydroxy-4-oxochromen-7-yl]oxy-3,4,5-trihydroxyoxane-2-carboxylic acid can be used as a candidate inhibitor for targeting Mpro [91]. Houttuynia cordata compound 7-oxodehydroasimilobine, (1,2,3,4,5-pentamethoxy-dibenzo-quinolin-7-one) and (1,2-dimethoxy-3-hydroxy-5-oxonoraporphine) widely produced in East Asia Since it can be docked with Rdrp to form a stable protein-ligand complex, it may be a promising drug candidate for SARS-CoV-2 infection [92]. Herbal drug and pharmacological components with anti-COVID-19 potential are summed in Table 2.

**Table 2.** Summary of herbal drug and components with anti-COVID-19 potential

Herbal plant	Compound	Chemical structure	Target	Reference
/	Kurarinone	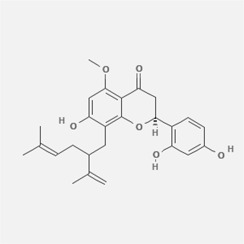	/	[81]
/	Artemisinin	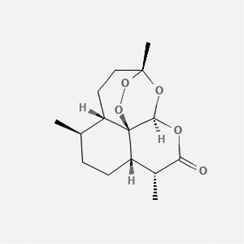	/	[93]
Rhizoma Polygonati	Diosgenin	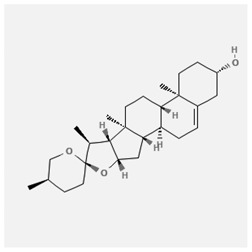	/	[94]
	(+)-Syringaresinol-O-beta-D-glucoside	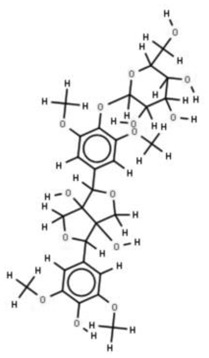		
Huang Qi, Fu Ling, Bai Zhu, Di Huang, Shan Yao	Quercetin	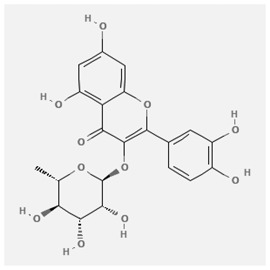	PTGS2 (COX2), PTGS1 (COX1), IL6, CASP3, NOS2, and TNF, etc.	[82]
	Formononetin	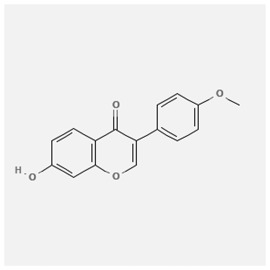		
	Kaempferol	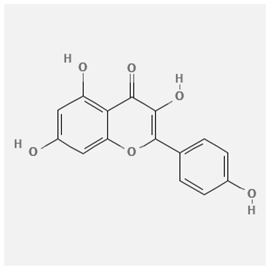		
Anupana	Epicatechin	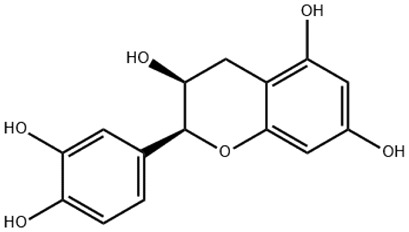	/	[98]
	Hesperidin	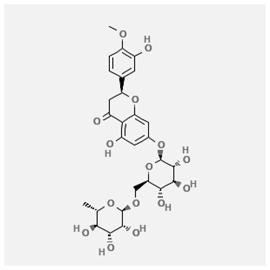		
/	Uncaria Tomentosa	/	ACE-2	[95]
Lianhua Qingwen and Shuanghuanlian	Rutin	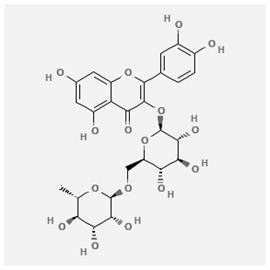	Mpro	82
Shufeng Jiedu	Quercetin	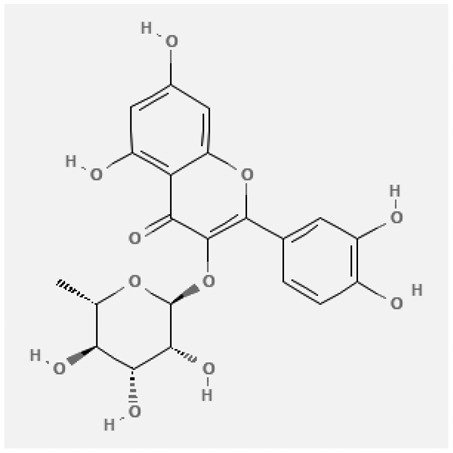	/	[97]
	Wogonin	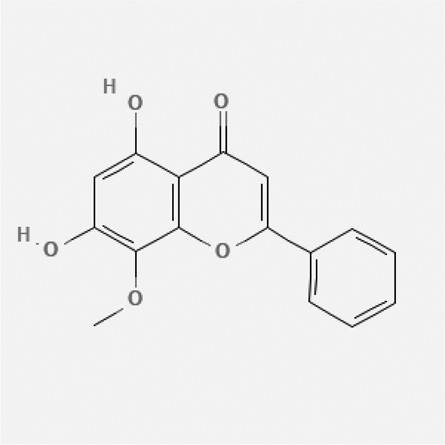		
	Polydatin	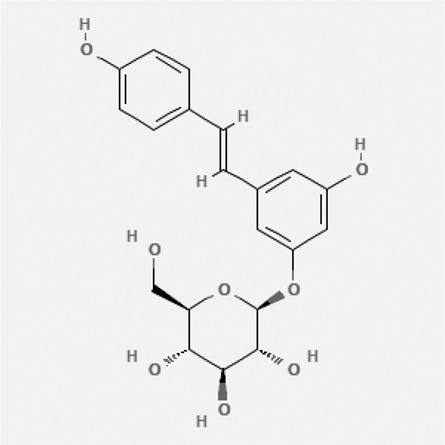		
Andrographis paniculata	/	/	Toll-like receptor pathway, PI3/AKT pathway and MAP kinase pathways	[52]
Piper nigrum	Moupinamide	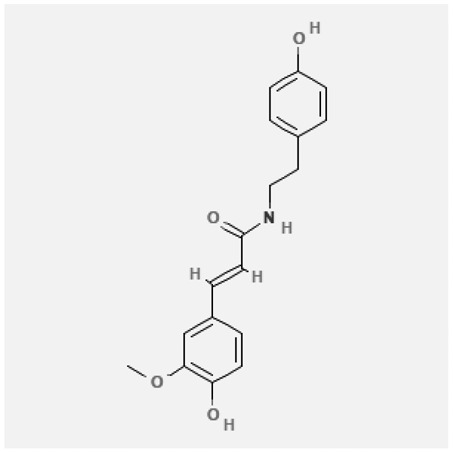	S Protein	[83]
EGYVIR extract (Curcuma longa root, Piper nigrum seeds)	Pentatriacontane	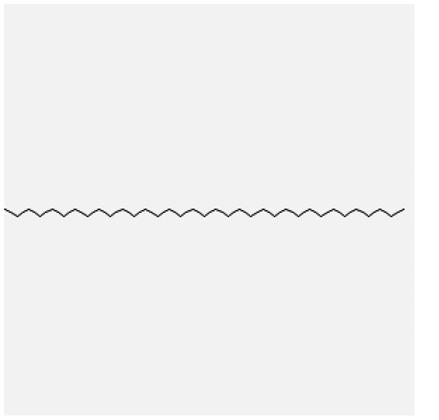	Spike receptor-binding domain, NF-κB pathway	[84]
	Curcumin	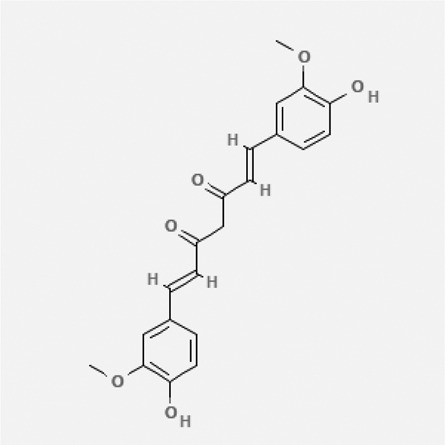		
	Piperine	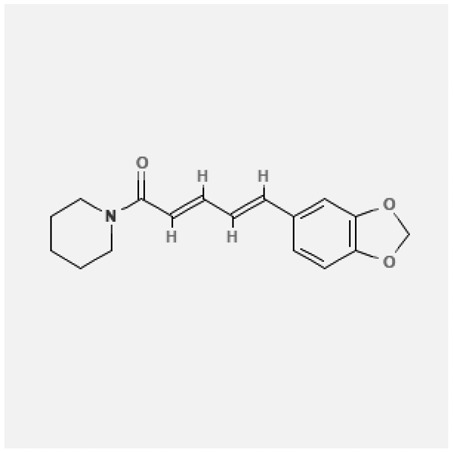		
	Sitosterol	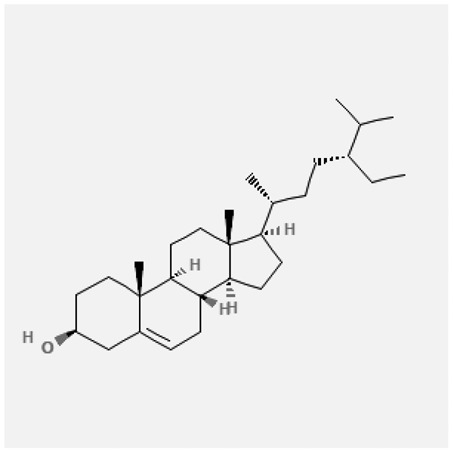		
	Amyrin	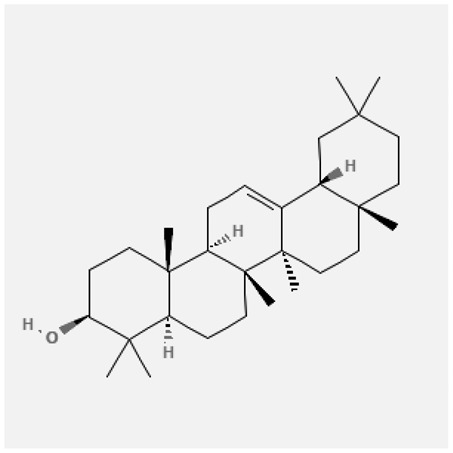 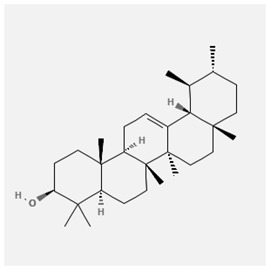		
	Turmerone	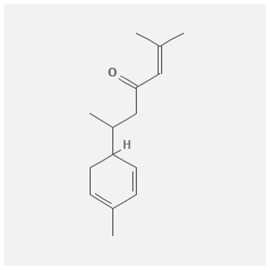		
	Lupeol	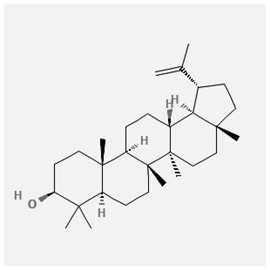		
	Quercetin	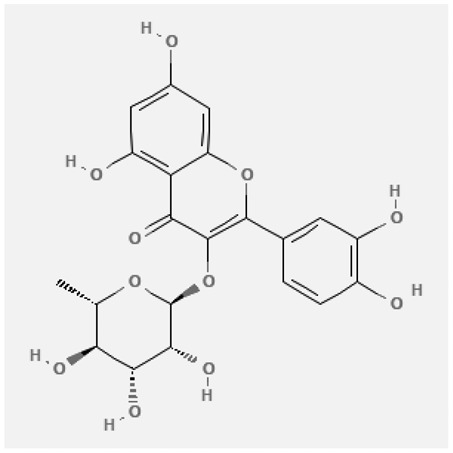	S Protein	[85]
Yangyinjiedu, Dayuanxiaodu and Chaihuqingzao	Epigallocatechin-3-gallate	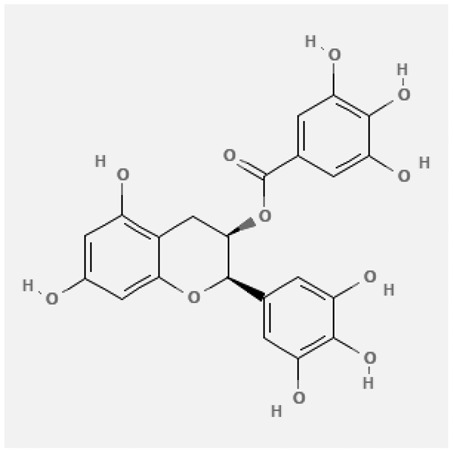	M_PRO_	[87]
Nigella sativa	Campesterol	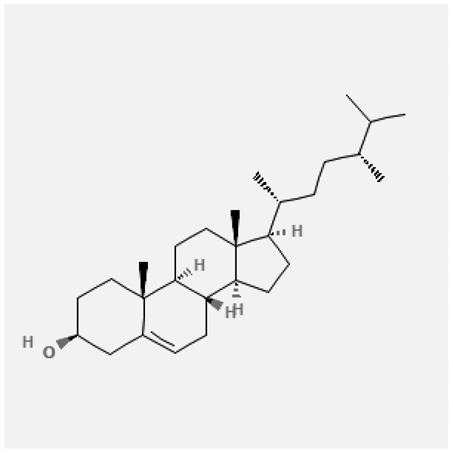	N and PL_PRO_	[86]
	Cycloeucalenol	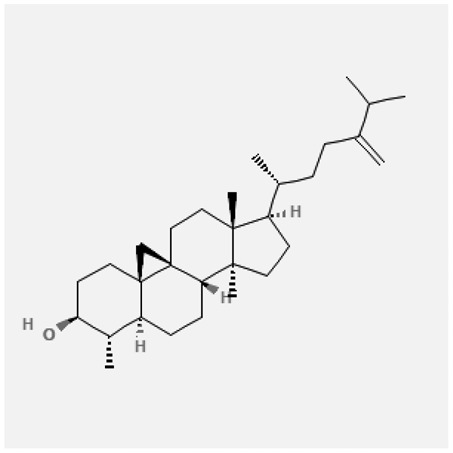		
	Alpha-spinasterol	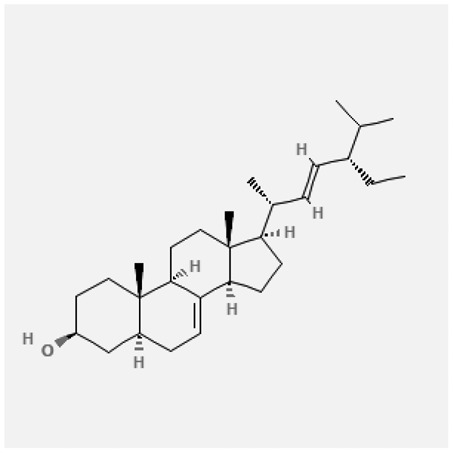		
	Beta-sitosterol	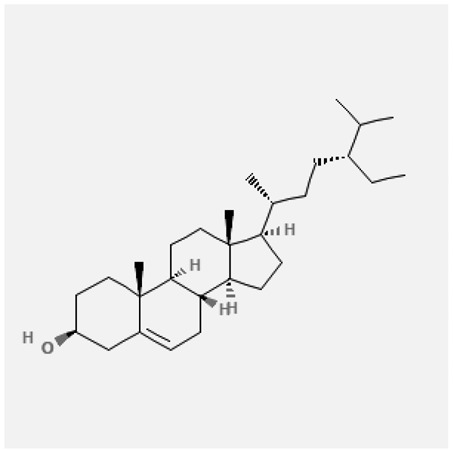		
	Cichoriin	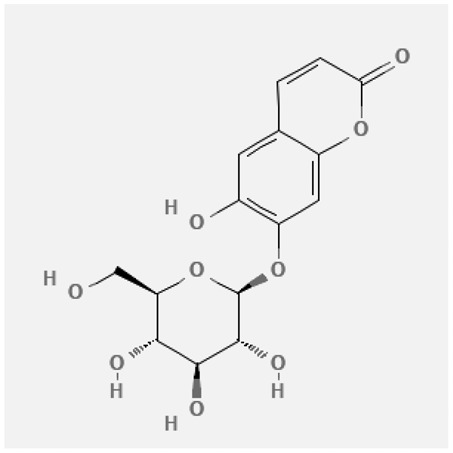	RdRp, Nsp14, Nsp3 and PL_PRO_	[62]
Ephedra sinica	Ephedrine	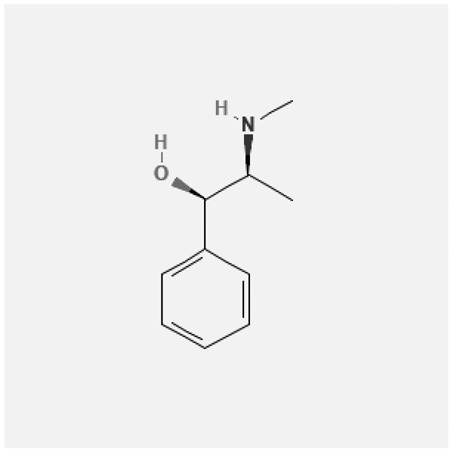	ACE2	[88]
	Pseudoephedrine	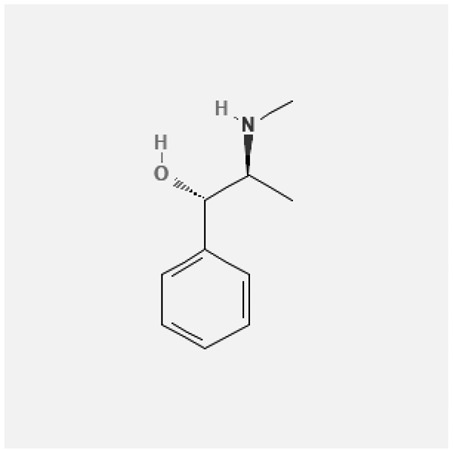		
	Methylephedrine	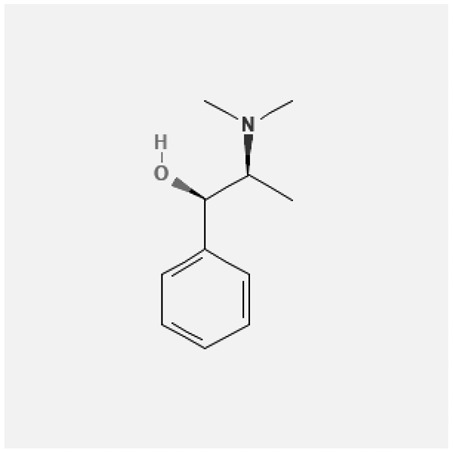		
	Eugenin	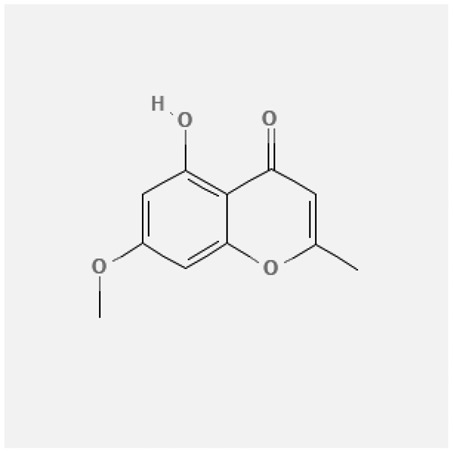	Several active site of the SARS-CoV-2	[50]
	Silymarin	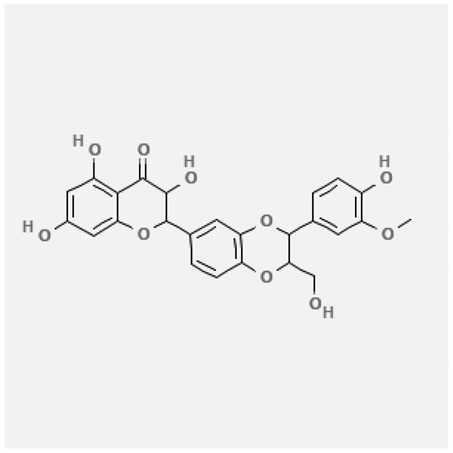		
	Amoxycillin	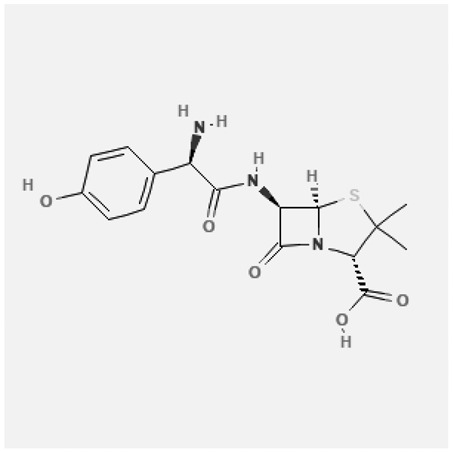		
	Curcumin	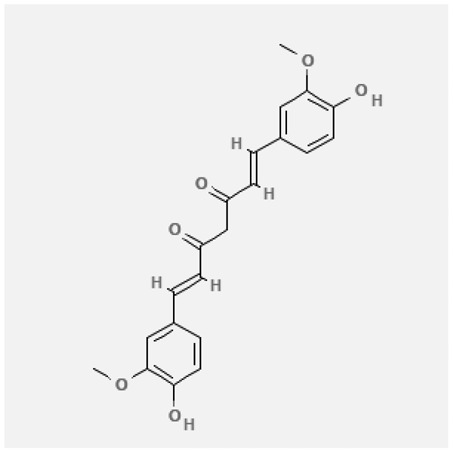		
Coronil	/	/	inhibiting spike protein-ACE-2 interactions	[99]
species found in the Brazilian herbal medicine	Triterpenes and Phenolic compounds	/	/	[89]
Horchata infusion	Benzoic Acid	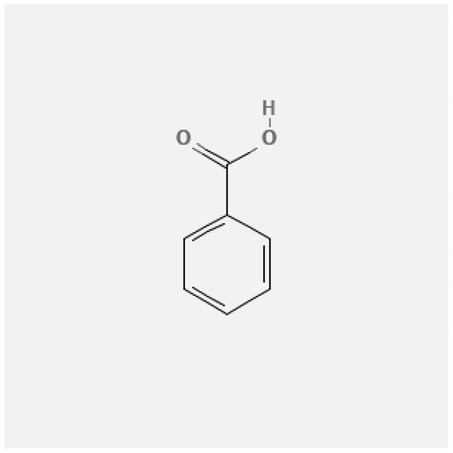	M_PRO_	[91]
	2-(ethylthio)-ethylester	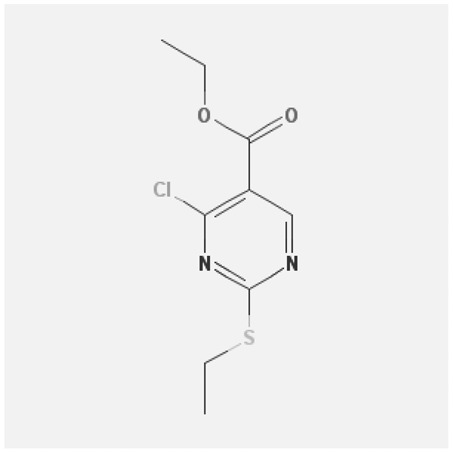		
	l-Leucine-N-isobutoxycarbony l-N-methyl-heptyl	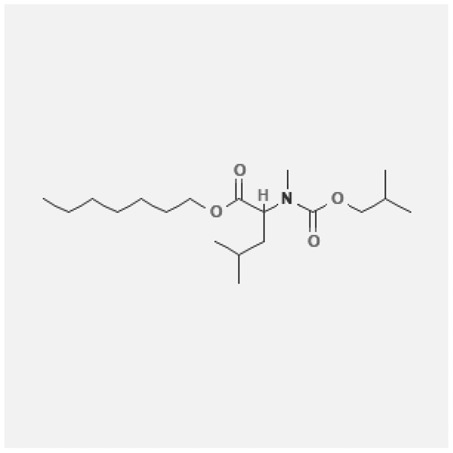		
	Isorhamnetin	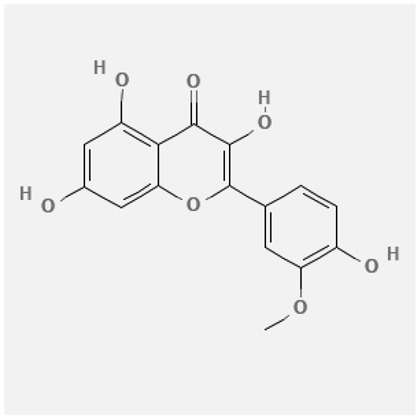		
	PLpro: isorhamnetin-3-O-(6-Orhamnosyl-galactoside)	/		
	dihydroxy-methoxyflavanone and dihydroxyphenyl)-5-hydroxy-4-oxochromen-7-yl]oxy-3,4,5-trihydroxyoxane-2-carboxylic acid	/		
*Houttuynia cordata* Thunb	7-oxodehydroasimilobine	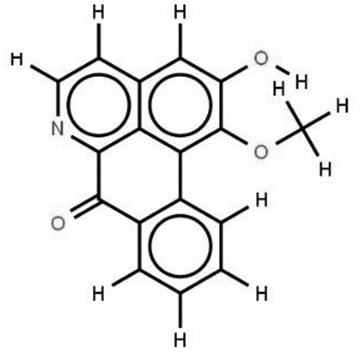	RdRp	[92]
	1,2,3,4,5-pentamethoxy-dibenzo-quinolin-7-one	/		
	1,2-dimethoxy-3-hydroxy-5-oxonoraporphine	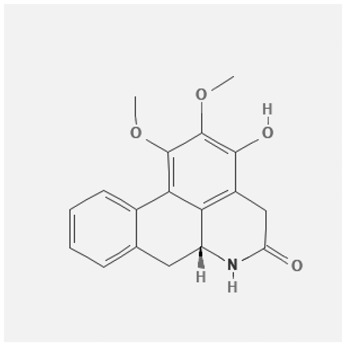		

In vitro prediction models show that artemisinin B and benzofluorenol, which have anticancer, antiviral and immunomodulatory effects, also have potential antiviral treatment prospects [93]. The molecular docking results showed that the compound saponin and (+)-Syringaresinol-O-beta-D-glucoside (diosgenin and (+)-Syringaresinol-O-beta-D-glucoside) in Rhizoma Polygonati and three SARS-CoV-2 protein has higher affinity. Therefore, the compounds in Polygonatum may act on different targets through virus and cancer-related signal transduction, and have great potential in the treatment of COVID-19 [94]. Computer-based molecular docking and simulation analysis showed that a group of natural spices and herbal secondary metabolites, such as epicatechin, embelin, cafestol, hesperidin, murrayanine and murrayaquinone-A, have potential antiviral effects. Molecular simulation results of Andres et al. found that Uncaria tomentosa components can bind to the ACE-2 site of SARS-CoV-2 RBD virus, which has potential antiviral properties and effectiveness as a supplement and/or alternative drug for COVID-19 treatment [95]. Rutin is a key ingredient in many herbs (such as LHQW and Shuanghuanglian). Molecular docking analysis shows that it can bind to the active site of the Mpro protein of SARS-CoV-2 to inhibit its biological function, and target Mpro inhibitor may have a drug development strategy to become an antiviral treatment [95,96]. In addition, the active ingredients of quercetin, wogonin, and polydatin in the TCM SFJD are also predicted to bind directly to Mpro, the main protease of SARS-CoV-2 [97]. Computer-based analysis by Saraswat et al. found that Syringin, Hesperetin, Silymarin, Curcumin, and Myricetin from natural plant species exhibit the largest combination of SARS-CoV-2 protease complex compounds, indicating that they can be used as anti-SARS-CoV-2 preparations [50].

S Protein, spike Protein; M_PRO_, chymotrypsin-like protease; RdRp, RNA-dependent RNA polymerase; PL_PRO_, papain-like protease; Nsp, non-structural proteins.

## 6. Effect and potential mechanism of herbal medicines in COVID-19 treatment

### 6.1 Effect of herbal medicines on COVID-19 treatment

Through the silico and biological processing, a series of small molecules, including those from natural compounds, have been screened and confirmed to directly inhibit these important proteins in SARS or MERS coronavirus [100–105]. The gene sequence of SARS-CoV-2 suggests high similarities with virus that previously identified in SARS-CoV or MERS-CoV [66]. In this sense, previously reported anti-SARS-CoV or anti-MERS-CoV natural compounds may be a valuable guide to finding anti-SARS-Cov-2 herbal plants among the traditional Chinese herbs used to treat viral pneumonia. Chinese herb formulaes used to treat SARS-CoV infection include Yin Qiao San [106,107], Yu Ping Feng San [108], Sang Ju Yin, LHQW Capsule [108], Shuanghuanglian [109], Ma Xin Gan Shi Tang [28] et al. These drugs have antiviral activity, anti-inflammatory and immune regulation functions, and can improve the function of upper respiratory mucosal immune system after administration. These drugs originally used for the treatment of SARS-CoV continue to be used for the treatment of COVID-19 this time. Among them, LHQW is recommended by the new guidelines for patients in the medical observation period. Clinical evidences suggest that LHQW capsules have a certain effect on obviously alleviating the symptoms of cough, fever, and fatigue in patients with COVID-19, reducing the proportion of severe cases, and shortening fever time and inhibiting the virus replication [110–114]. There is also evidence that Shuanghuanglian, a Chinese herbal product made of honeysuckle and forsythia, can inhibit the activity of SARS-CoV-2 [115]. Similarly, LHQW, composed of 13 herbs and 733–3084 chemical ingredients also shows antiviral activity and is capable of inhibiting the replication of SARS-CoV-2. Further research shows that it may be related to regulating the human immune response and reducing the cytokine storm [116,117]. Chinese medicine QFPD, which is recommended for COVID-19 treatment in China, has a potential role of inhibiting entry and replication of SARS-CoV-2 by targeting the 3CLpro protein and host ACE2 [116,117]. SFJD, consisting of eight medicinal herbs, is widely used for COVID-19 for its antiviral, antibacterial, antitumor effects, and anti-inflammatory activities [118]. A clinical real-world pragmatic study has shown that compared with standard treatment, the number of days of fatigue and coughing symptoms in patients with combined use of SFJD has been significantly reduced. Provided SFJD be used as early as possible in the first 8 days once the onset of symptoms, the benefits of patients shall be more obvious [97]. Yindan Jiedu Granules (YDJDG) is a Chinese herbal formula newly applied by Beijing Ditan Hospital. Compared with lopinavir-ritonavir treatment, YDJDG can shorten the average duration of fever and lung exudative lesions to speed up [119]. Shenhuang Granule (SHG), composed of ginseng, palm rhubarb stem, sea cucumber, dandelion, aconite, and Whitmania pigra Whitman, has a significant effect on shortening the hospital stay of severely ill patients [120]. Recently, Brian et al. reported an oral liquid of traditional medicine, respiratory Detox Shot, which contains mostly herbal ingredients against traditional lung diseases, can directly inactivates the infectivity of SARS-CoV-2 virus particles, and inhibited the infection of lentivirus based lenti-SARS-CoV, lenti-SARS-CoV-2 [121]. In summary, clinical trials and in vitro evidence have suggested that Chinese medicine granules as a whole is playing a role in reducing mortality and reducing fever [122,123]. However, these clinics are not standard randomized clinical trials, and further large-scale randomized, double-blinded, placebo-controlled clinical trials are needed to provide more confident evidence. Nigella sativa is a famous edible spice with antiviral and immunomodulatory activities, which was used in an open-label randomized controlled clinical trial of Saudi Arabia to reduce symptoms and shorten the recovery time of symptomatic patients with mild COVID-19 [45]. Another open-label randomized controlled clinical trial from India showed that Kabasura Kudineer reduced the SARS-CoV-2 viral load of asymptomatic COVID-19 patients and did not record any adverse reactions [54].

### 6.2 Herbs in combination with conventional therapy in COVID-19 treatment

In addition, superiority was displayed in the treatment of COVID-19 through a combination of the Huashi baidu (HSBD) Formula [54]. Compared with treatment with western medicine alone, TCM combined with western medicine can benefit patients more by reducing the rate of exacerbation of the disease. At the same time, there is no increase in the incidence of adverse events [124]. Liu et al. observed that Arbidol combined with LHQW treatment shortened the time for the negative result of the SARS-CoV-2 nucleic acid detection, and had a better effect on the resolution of lung inflammation than LHQW alone [125]. Another clinical trial showed that LHWQ combined with conventional treatment can shorten fever, fatigue and cough and recovery time than conventional treatment, with better effects on improving chest CT performance and clinical cure rate [116].

In addition to Chinese herbal formulas, traditional Chinese medicine injections also have immunosuppressive function. Wang et al. reported that Shen Fu Injectio, Shenmai Injection and Re Du Ning Injection could inhibit the levels of inflammatory factors such as IL-1β, IL-6, TNFα and other factors, thereby reducing the function of lung inflammation [126–129]. Xiyanping injection can significantly improve the symptom relief and virus clearance time of patients with mild to moderate COVID-19 [130]. Therefore, it can be speculated that the mechanism of traditional Chinese medicine injections in the treatment of COVID-19 may be related to the reduction of tissue inflammation levels. Future experiments on cell and animal levels will provide more detailed information.

### 6.3 Potential mechanism of herbal medicines in COVID-19 treatment

#### 6.3.1 Disturb SARS-CoV-2 replication and proliferation

Drug targets to intervene based on key molecules or events at each stage of the virus life cycle is a strategy for many drug developments. In the life cycle of SARS-CoV-2, the S protein is the main protein for the virus to invade the host. It is also a key factor in determining the host tropism and tissue phagocytosis of the coronavirus. Previous studies have found that mutations in the SARS-CoVS protein, such as N479L and T487S, can significantly increase the affinity of the virus to human ACE2, while mutations in the MERS-CoV S protein may contribute to the virus zoonotic Sexual transfer [131], which shows that S protein and host are very important for the establishment of coronavirus infection in humans. The ORF1ab protein occupy two-thirds of the viral genome can be cleaved into at least 16 predicted nsps [132]. Among them, PLpro protein protease and phosphatase activities, participating in regulating the virus replication process and has the function of antagonizing IFN antagonism [133], and 3CLpro and Rdrp regulate RNA replication [134]. Therefore, S protein and ORF1ab protein have become the main targets of anti-SARS-CoV-2 drugs. Conversely, the inhibition of virus entry into cells, replication, and translation is also one of the possible mechanisms for many drugs to treat COVID-19. QFPD decoction is reported to directly inhibit the viral invasion and replication in the treatment of COVID-19 [135]. For the study of anti-SARS-CoV-2 drug mechanism, the structure-based screening of chemical or phytochemical databases can provide clues to large numbers of crystal target [136]. A prediction based on molecular simulation showed that the ingredients in Chinese medicine prescriptions such as quercetin [137], andrographolide [138], glycyrrhizic acid [139], baicalin [139,140], patchouli alcohol [141], and luteolin [142] had binding sites against SARS-CoV-2 protein, 3CLpro, ACE2, S protein, Rdrp and PLpro. In addition, their potentially role in the interference of viral life cycles have been previously reported that these components may be able to inhibit virus infection by binding to SARS-CoV-2 protein, inhibiting virus entry into cells, inhibiting the activation of virus-induced cellular pathways, and inhibiting virus replication and proliferation in the body. An analysis of the protein-protein interaction core network using the TCM Systems Pharmacology Database and Analysis Platform study suggested that there are many COVID-19-related genes acted by the active Chinese herbal medicines ingredients such as 9,10-dimethoxypterocarpan-3-O-b-D-glucoside [143]. Ye et al. speculated that rutin, forsythoside E, and hyperoside components in LHQW may directly target to main protease of SARS-CoV-2 and exert anti-virus effects [144,145]. Chen et al. study showed that baicalin, scutellarin, hesperetin, glycyrrhizin and nicotianamine could interact with ACE2, and Chinese herbs contain these compounds may have a potential role in antiviral invasion and infection []. The molecular interaction analysis of QFPD components shows that QFPD may directly inhibit virus invasion and replication. Among them, Pogostemonis Herba, Bupleuri Radix, Polyporus, Asteris Radix et Rhizoma, 23-acetic acid alisol B can directly target the SARS-CoV-2 3CL pro protein to prevent virus proliferation. Alcohol, Farfarae Flos, ergosterol, asaramine, Ephedrae Herba and Asteris Radix et Rhizoma in QFPD have ACE2 binding sites; it may block SARS-Cov-2 from binding to host ACE2 and affect virus invasion of the epithelium cell [,^146^]. Sun et al. found that baicalein and licorice phenol in HSBD formula may target Mpro and ACE2 proteins []. The active ingredients of JHQG granules Kaempferol, quercetin, luteolin, baicalein, oroxylin A,licochalcone B, and glyasperin C have the potential to target ACE2, and may regulate PTGS2, BCL2, CASP3 and related pathways, thereby affecting virus replication [147,148,149]. Herbal medicines also function as suppressor of stress and anxiety and improve mental health in the context of COVID-19 [150]

#### 6.3.2 Affecting host immune/ inflammatory response

Studies based on imaging and pathology have shown that COVID-19 is not just a lung infection, but also has the manifestations of thrombotic inflammation. And with the progress of the disease, SARS-CoV-2 infection initially affects the lungs and perfuses organs through the whole body [151]. It is worth noting that there are many factors that affect the progression of the disease, besides the viral load, but also other factors such as poor interferon response [152]. It is currently believed that the inflammatory factor storm caused by the body’s excessive inflammatory response to SARS-CoV-2 infection is the main cause for the severity of disease and death of COVID-19 patients [153]. The high levels of circulating cytokines and lymphocytes in the lungs, heart, spleen, lymph nodes and kidneys and a large number of mononuclear cell infiltration may be closely related to the severity of COVID-19 disease [154]. A study of KEGG enrichment analysis of a series of 26 herbal components predicted to have binding sites on viral proteins showed that the pathways of these herbal actions were related to the regulation of viral infections, immune/inflammatory responses and hypoxia responses, suggesting the potential regulatory pathways of these herbs in viral respiratory infections [155]. In vitro cell experiments and virological experiments showed that LHQW, which is widely used to treat COVID19, not only significantly inhibited the replication of SARS-CoV-2 in Vero E6 cells, but also reduced the expression of pro-inflammatory cytokines (TNF-α, IL-6, CCL-2/MCP-1 and CXCL-10/IP-10) [155]. Moreover, chemical components of LHQW may inhibit inflammation-related pathways such as JAK-STAT, PI3K-AKT, MAPK, and NF-κB, etc. [25,156]. QFPD another Chinese medicine formula widely used in COVID-19 treatment [157]. QFPD has more than 300 active ingredients and can act on more than 790 targets [158]. Emerging evidences has revealed that QFPD may inhibit the invasion and replication of SARS-CoV-2 by directly targeting the virus 3C protein and host ACE2 [159], whose main target organ is the lung, followed by the spleen. By regulating a series of proteins co-expressed with ACE2 and a series of signal transduction pathways closely related to the occurrence and development of diseases, QFPD plays a role in balancing immunity and eliminating inflammation, inhibiting the translation of viral mRNA and inhibit a group of proteins that interact with viral proteins by targeting ribosomal proteins (ribosomal proteins necessary for virus replication) [157]. GO enrichment and KEGG pathway annotation analysis suggest that IL6, IL10, MAPK, CXCL8, CCL2, IL1B, and PTGS2 genes are potentially targets of compounds of Qingfeng Jiedu granules [160]. Ren reported that the frequently used Chinese formulas for COVID-19, including HXZQ capsules, JHQG granules, LHQW capsules, QFPD decoction, play a protective role against SARS-CoV-2 via regulating cytokines levels through the arachidonic acid metabolic pathway [161]. For SFJD, several studies suggest its active ingredients that might be involved in anti-inflammation. These ingredients (quercetin, β-sitosterol, luteolin, acacetin, wogonin, kaempferol, licochalcone A, andisorhamnetin, 5,7,4’-trihydroxy-8-methoxyflavone) might target on inflammatory cytokine (IL6, IL1B, CCL2, MAPK8, MAPK1, MAPK14, CASP3, FOS, ALB, CALM1, NOS2, PTGS2, DPP4, and PTGS2) and signaling pathways [127,162–164]. Yang et al. analyzed the transcriptomic profile of ILP induced pneumonia and found that Toll-like signaling pathway might be one of the most important pathways for Qingfeng Paidu Decoction that mediated anti-SRARS-CoV-2 effect, and inhibition of Toll-like signaling pathway agonist and IL-6 production in macrophage involved in the antiviral mechanism of MXSG [165]. Notably, Chinese medicine is to targets diversity, and some Chinese medicines have been found to have the functions of inhibiting virus replication and anti-inflammatory. For example, Liu Shen capsule SARS-CoV-2 replication in Vero E6 cells, prevent and inhibits the mRNA level of TNF-α, IL-6, IL-1β, IL-8, CCL-2/MCP-1 and CXCL-10/IP-10 [166].

A pilot randomized clinical trial revealed that Xuanfei Baidu decoction can not only increase white blood cells and lymphocytes, but also reduce C-reactive protein and erythrocyte sedimentation rate [167]. Tanreqing Capsule is a TCM. A retrospective cohort study showed that the use of Tanreqing Capsule as a conventional drug supplement can benefit patients with mild and moderate COVID-19 and increase the level of immune CD3 + T cells in patients [168]. The herbal formulation Divya-Swasari-Vati contains a mixture of different plant compounds, such as gallic acid, ellagic acid, cinnamic acid, eugenol, 6-gingerol, piperine and glycyrrhizin, which can reduce spike protein-induced inflammation in humanized zebrafish model [53].

Herbal medicine also has a therapeutic effect on COVID-19-related complications. The study found that Arenaria kansuensis scientifically alleviated pulmonary fibrosis caused by COVID-19. The possible pathway is to activate Nrf2 and inhibit NF-kB/TGF-β1/Smad2/3 pathway [169]. In addition, the analysis of the drug network topology suggests that the TCM for rehabilitation can inhibit fibrosis-related signal pathways such as VEGF, MAPK and TGF-β1 [53,170].

## 7. Limitations

Although Chinese medicine is used for antiviral, especially for the treatment of COVID19, and sufficient evidence has also shown its role in patients’ symptoms, there is a concern that it is difficult to determine whether the antiviral activity of a CHM is a consequence of a single and precise action mechanism or a result of a synergistic therapeutic effect. Unable to provide high supply, may be also the Challenge faced by implementing Chinese medicine in worldwide application for Covid-19 treatment. In addition, a significant limitation is the lack of rigorous randomized controlled trial demonstrations, which has raised a major concern on safety [171]. Therefore, future prospective cohort studies, RCTs or registry studies as well as bioinformatics-based target analysis to evaluate the effectiveness of herbal medicine formulas in preventing COVID-19 are to conduct to provide stronger evidence for the application of herbal medicines to prevent COVID-19 and the treatment of re-explosive conditions to maximize the benefits of patients with indications.

## 8. Conclusion

Collectively, due to the lack of full knowledge of pathology of COVID, the complexity of the pathological mechanisms of the above-mentioned diseases suggests that single-target drug development has its limitations. The various components of traditional medicine prescriptions may target multiple therapeutic targets signaling and host cell cytokine signaling, making it possible to play a unique role in the treatment of COVID-19. Now herbal medicine has been widely used against COVID-19 in mainland China, East Asian, Saudi, Arabia, Bangladesh, Moroccan, Indian, Africa, America. In this review, we have summarized the application of herbal medicine in prevention and treatment of corona viruses, antiviral activity ingredients of herbal medicine, and the progress of related molecular mechanism of herbal medicine. Especially, we took a simple outline of the application in the fight against COVID-19 in China. In addition, we suggested that Kurarinone, Anupana containing epicatechin and hesperidin, curcumin and piperine have protection against epithelial damage caused by virus infection and anti-coronavirus anti-infective activity. We also propose that current herbal medicines mainly improve or treat COVID-19 by inhibiting virus entry, replication, or improving the host’s immune defense level. Moreover, in the treatment of COVID-19, it may be beneficial to use herbal medicines in combination with Western medicine although these need to be supported by subsequent large-scale clinical trials.

1. Perspective

Taking into account public health emergency, these alternative treatment options of herbal medicine may expand the thinking and provide a variety of options for symptom relief and treatment of unsuspected patients and ones to be confirmed, and provide the possibility of personalized treatment for COVID-19. Herbal medicine could be used as a supplement to western medicine in the world, and it can encourage and inspire more countries finding new herbal medicines to prevent and treat the new coronavirus. In the future, according to their own health and economic conditions, a variety of new crown prevention and control programs will be launched.

## Highlights

- Usage of herbal medicine in COVID-19 prevention and treatment in mainland China
- Herbal medicine used for COVID-19 prevention and treatment worldwide.
- Potential mechanism of herbal medicines in COVID-19 prevention and treatment.

## References

[1] China National Health Commission. Diagnosis and treatment of pneumonitis caused by new coronavirus (trial version 7). In.: Beijing China National Health;2020. http://www.nhc.gov.cn/jkj/s3577/202009/318683cbfaee4191aee29cd774b19d8d.shtml

[2] Zu ZY, Jiang MD, Xu PP, et al. Coronavirus disease 2019 (COVID-19): a perspective from China. Radiology. 2020;296(2):E15–E25. DOI:10.1148/radiol.2020200490. 32083985 PMC7233368

[3] Dong Y, Mo X, Hu Y, et al. Epidemiology of COVID-19 among children in China. Pediatrics. 2020;145(6). DOI:10.1542/peds.2020-0702. 32179660

[4] Liu Y, Eggo RM, Kucharski AJ. Secondary attack rate and superspreading events for SARS-CoV-2. Lancet. 2020;395(10227):e47. 32113505 10.1016/S0140-6736(20)30462-1 PMC7158947

[5] Lu X, Zhang L, Du H, et al. SARS-CoV-2 infection in children. New Engl J Med. 2020;382(17):1663–1665. DOI:10.1056/NEJMc2005073. 32187458 PMC7121177

[6] Ibarrondo FJ, Fulcher JA, Goodman-Meza D, et al. Rapid decay of Anti-SARS-CoV-2 antibodies in persons with mild Covid-19. N Engl J Med. 2020;383(11):1085–1087. DOI:10.1056/NEJMc2025179. 32706954 PMC7397184

[7] Zhu FC, Guan XH, Li YH, et al. Immunogenicity and safety of a recombinant adenovirus type-5-vectored COVID-19 vaccine in healthy adults aged 18 years or older: a randomised, double-blind, placebo-controlled, phase 2 trial. Lancet. 2020;396(10249):479–488. DOI:10.1016/S0140-6736(20)31605-6. 32702299 PMC7836858

[8] Dagotto G, Yu J, Barouch DH. Approaches and Challenges in SARS-CoV-2 vaccine development. Cell Host Microbe. 2020;28(3):364–370. 32798444 10.1016/j.chom.2020.08.002 PMC7416703

[9] Susskind D, Vines D. The economics of the COVID-19 pandemic: an assessment. Oxford Rev Econ Pol. 2020;36(Supplement_1):S1–S13. 10.1093/oxrep/graa036 PMC7499690 40504180

[10] Jin YH, Cai L, Cheng ZS, et al. A rapid advice guideline for the diagnosis and treatment of 2019 novel coronavirus (2019-nCoV) infected pneumonia (standard version). Mil Med Res. 2020;7(1):4. DOI:10.1186/s40779-020-0233-6. 32029004 PMC7003341

[11] Han YY, Zhao MR, and Shi Y, et al. Application of integrative medicine protocols on treatment of coronavirus disease 2019. Chin Tradit Herb Drugs. 2020;2020:2–18.

[12] China National Health Commission. Diagnosis and treatment of pneumonitis caused by new coronavirus (trial version 6). In.; 2020.

[13] Xiao M, Tian J, Zhou Y, et al. Efficacy of Huoxiang Zhengqi dropping pills and Lianhua Qingwen granules in treatment of COVID-19: a randomized controlled trial. Pharmacol Res. 2020;161:105126. 32781283 10.1016/j.phrs.2020.105126 PMC7414728

[14] Province S. Clinical features and treatment of COVID‐19 patients in northeast Chongqing. 10.1002/jmv.25783 PMC7228368 32198776

[15] Hung I, To K, Lee CK, et al. Hyperimmune IV immunoglobulin treatment: a multicenter double-blind randomized controlled trial for patients with severe 2009 influenza A(H1N1) infection. Chest. 2013;144(2):464–473. DOI:10.1378/chest.12-2907. 23450336

[16] Zhu YL, Yang BB, Wu F. Understanding of COVID-19 in children from different perspectives of traditional Chinese medicine and western medicine. Chinese Traditional and Herbal Drugs. 2020. 51(4)

[17] Ni L, Zhou L, Zhou M, et al. Combination of western medicine and Chinese traditional patent medicine in treating a family case of COVID-19. Front Med. 2020;14(2):210–214. DOI:10.1007/s11684-020-0757-x. 32170559 PMC7088740

[18] Chan KW, Wong VT, Tang SCW. COVID-19: An update on the epidemiological, clinical, preventive and therapeutic evidence and guidelines of integrative Chinese–Western medicine for the management of 2019 novel coronavirus disease. Am J Chin Med. 2020;48(3):737–762. 32164424 10.1142/S0192415X20500378

[19] Mohamad Hesam ShahrajabianMohamad Hesam Shahrajabian Biotechnology Research Institute CAOA. A review of ginseng species in different regions as a multipurpose herb in traditional Chinese medicine, modern herbology and pharmacological science. J Med Plants Res. 2019;10(13):213–226.

[20] Sun W, Shahrajabian MH, Cheng Q. Anise (Pimpinella anisum L), a dominant spice and traditional medicinal herb for both food and medicinal purposes. Cogent Biology. 2019;5(1):1673688. 10.1080/23312025.2019.1673688

[21] Xia S, Zhong Z, and Gao B, et al. The important herbal pair for the treatment of COVID-19 and its possible mechanisms. Chin Med. 2021;16(1): 1–16 33658066 10.1186/s13020-021-00427-0 PMC7927769

[22] Chia WY, Kok H, Chew KW, et al. Can algae contribute to the war with Covid-19?. Bioengineered. 2021;12(1):1226–1237. DOI:10.1080/21655979.2021.1910432. 33858291 PMC8806238

[23] Peter AP, Wayne CK, Show PL, et al. Potential pathway that could treat Coronaviruses (COVID-19). Current Biochemical Engineering. 2020;6(1):3–4. DOI:10.2174/2212711906999200228100507

[24] Yuan H, Ma Q, Ye L, et al. The traditional medicine and modern medicine from natural products. Molecules. 2016;21(5):559. DOI:10.3390/molecules21050559. PMC6273146 27136524

[25] Tong T, Wu YQ, Ni WJ, et al. The potential insights of traditional Chinese medicine on treatment of COVID-19. Chin Med. 2020;15(1):51. DOI:10.1186/s13020-020-00326-w. 32483409 PMC7245619

[26] Jiang W. Therapeutic wisdom in traditional Chinese medicine: a perspective from modern science. Trends Pharmacol Sci. 2005;26(11):558–563. 16185775 10.1016/j.tips.2005.09.006

[27] Miao Q, Cong XD, Wang B, et al. Understanding and thinking of novel coronavirus pneumonia in traditional Chinese medicine. J Trad Chin Med. 2020;61. 286–288.

[28] Ang L, Lee HW, Choi JY, et al. Herbal medicine and pattern identification for treating COVID-19: a rapid review of guidelines. Integr Med Res. 2020;9(2):100407. DOI:10.1016/j.imr.2020.100407. 32289016 PMC7104236

[29] Lau TF, Leung PC, Wong EL, et al. Using herbal medicine as a means of prevention experience during the SARS crisis. Am J Chin Med. 2005;33(3):345–356. DOI:10.1142/S0192415X05002965. 16047553

[30] Needham J, Gwei-djen L. Hygiene and preventive medicine in ancient China. J Hist Med Allied Sci. 1962;17(4):429–478. 13937874 10.1093/jhmas/xvii.4.429

[31] Luo H, Tang QL, Shang YX, et al. Can Chinese medicine be used for prevention of Corona virus disease 2019 (COVID-19)? A review of historical classics, research evidence and current prevention programs. Chin J Integr Med. 2020;26(4):243–250. DOI:10.1007/s11655-020-3192-6. 32065348 PMC7088641

[32] Du CY, Zheng KY, Bi CW, et al. Yu Ping Feng San, an Ancient Chinese herbal decoction, induces gene expression of anti-viral proteins and inhibits neuraminidase activity. Phytother Res. 2015;29(5):656–661. DOI:10.1002/ptr.5290. 25586308

[33] Hoever G, Baltina L, Michaelis M, et al. Antiviral activity of glycyrrhizic acid derivatives against SARS-coronavirus. J Med Chem. 2005;48(4):1256–1259. DOI:10.1021/jm0493008. 15715493

[34] Wang C, Cao B, Liu QQ, et al. Oseltamivir compared with the Chinese traditional therapy maxingshigan-yinqiaosan in the treatment of H1N1 influenza: a randomized trial. Ann Intern Med. 2011;155(4):217–225. DOI:10.7326/0003-4819-155-4-201108160-00005 21844547

[35] Pu J, Mei H, Lei L, et al. Knowledge of medical professionals, their practices, and their attitudes toward traditional Chinese medicine for the prevention and treatment of coronavirus disease 2019: a survey in Sichuan, China. Plos One. 2021;16(3):e234855. DOI:10.1371/journal.pone.0234855. PMC7963037 33725021

[36] Ang L, Lee HW, Choi JY, et al. Herbal medicine and pattern identification for treating COVID-19: a rapid review of guidelines. Integr Med Res. 2020;9(2):100407. 32289016 10.1016/j.imr.2020.100407 PMC7104236

[37] Lee B, Lee JA, Kim K, et al. A consensus guideline of herbal medicine for coronavirus disease 2019. Integr Med Res. 2020;9(3):100470. DOI:10.1016/j.imr.2020.100470. 32691002 PMC7335489

[38] Kim D, Chu H, Min BK, et al. Telemedicine center of Korean medicine for treating patients with COVID-19: a retrospective analysis. Integr Med Res. 2020;9(3):100492. DOI:10.1016/j.imr.2020.100492. 32802745 PMC7395239

[39] Takayama S, Arita R, Ono R, et al. Treatment of COVID-19-related olfactory disorder promoted by kakkontokasenkyushin’i: a case series. Tohoku J Exp Med. 2021;254(2):71–80. DOI:10.1620/tjem.254.71. 34108344

[40] Takayama S, Namiki T, Ito T, et al. A multi-center, randomized controlled trial by the integrative management in Japan for epidemic disease (IMJEDI study-RCT) on the use of Kampo medicine, kakkonto with shosaikotokakikyosekko, in mild-to-moderate COVID-19 patients for symptomatic relief and prevention of severe stage: a structured summary of a study protocol for a randomized controlled trial. Trials. 2020;21(1):1–3. DOI:10.1186/s13063-019-3906-2. 33008479 PMC7530547

[41] Aomatsu N, Shigemitsu K, Nakagawa H, et al. Efficacy of Ninjin’yoeito in treating severe coronavirus disease 2019 in patients in an intensive care unit. Neuropeptides. 2021;90:102201. 34753072 10.1016/j.npep.2021.102201 PMC8484001

[42] Kuchta K, Cameron S, Lee M, et al. Which East Asian herbal medicines can decrease viral infections?. Phytochem Rev 2021 1–19 10.1007/s11101-021-09756-2 PMC8391007 34466134

[43] AlNajrany SM, Asiri Y, Sales I, et al. The commonly utilized natural products during the COVID-19 pandemic in Saudi Arabia: a cross-sectional online survey. Int J Environ Res Public Health. 2021;18(9):4688. DOI:10.3390/ijerph18094688. 33924884 PMC8125191

[44] ALkharashi NA. The consumption of nutritional supplements and herbal products for the prevention and treatment of COVID-19 infection among the Saudi population in riyadh. Clinical Nutrition Open Science. 2021;39:11–20. 10.1016/j.nutos.2021.09.001 34585172 PMC8461002

[45] Koshak AE, Koshak EA, Mobeireek AF, et al. Nigella sativa for the treatment of COVID-19: an open-label randomized controlled clinical trial. Complement Ther Med. 2021;61:102769. 34407441 10.1016/j.ctim.2021.102769 PMC8364675

[46] Ahmad A, Husain A, Mujeeb M, et al. A review on therapeutic potential of Nigella sativa: a miracle herb. Asian Pac J Tropical Biomedicine. 2013;3(5):337–352. DOI:10.1016/S2221-1691(13)60075-1. PMC3642442 23646296

[47] Rahman MT. Potential benefits of combination of Nigella sativa and Zn supplements to treat COVID-19. J Herbs Med. 2020;23:100382. 10.1016/j.hermed.2020.100382 PMC7313527 32834942

[48] Bouchentouf S, Missoum N. Identification of Compounds from Nigella Sativa as new potential inhibitors of 2019 novel Coronasvirus (Covid-19): molecular docking study. 2020.

[49] Ahmed I, Hasan M, Akter R, et al. Behavioral preventive measures and the use of medicines and herbal products among the public in response to Covid-19 in Bangladesh: a cross-sectional study. Plos One. 2020;15(12):e243706. DOI:10.1371/journal.pone.0243706. PMC7732085 33306725

[50] Saraswat J, Singh P, Patel R. A computational approach for the screening of potential antiviral compounds against SARS-CoV-2 protease: ionic liquid vs herbal and natural compounds. J Mol Liq. 2021;326:115298. 33518856 10.1016/j.molliq.2021.115298 PMC7832122

[51] Charan J, Bhardwaj P, Dutta S, et al. Use of complementary and alternative medicine (CAM) and home remedies by COVID-19 patients: a telephonic survey. Indian J Clin Biochem. 2021;36(1):108–111. DOI:10.1007/s12291-020-00931-4. PMC7602770 33162692

[52] Banerjee S, Kar A, Mukherjee PK, et al. Immunoprotective potential of ayurvedic herb kalmegh (Andrographis paniculata) against respiratory viral infections–LC–MS/MS and network pharmacology analysis. Phytochem Analysis. 2021;32(4):629–639. DOI:10.1002/pca.3011 33167083

[53] Balkrishna A, Verma S, Solleti SK, et al. Calcio-herbal medicine Divya-Swasari-Vati ameliorates SARS-CoV-2 spike protein-induced pathological features and inflammation in humanized zebrafish model by moderating IL-6 and TNF-α cytokines. J Inflamm Res. 2020;13:1219. 33414643 10.2147/JIR.S286199 PMC7783203

[54] Natarajan S, Anbarasi C, Sathiyarajeswaran P, et al. Kabasura Kudineer (KSK), a poly-herbal Siddha medicine, reduced SARS-CoV-2 viral load in asymptomatic COVID-19 individuals as compared to vitamin C and zinc supplementation: findings from a prospective, exploratory, open-labeled, comparative, randomized controlled trial, Tamil Nadu, India. Trials. 2021;22(1):1–11. DOI:10.1186/s13063-020-04976-x. 34526104 PMC8441246

[55] Dandara C, Dzobo K, Chirikure S. COVID-19 pandemic and Africa: from the situation in Zimbabwe to a case for precision herbal medicine. OMICS. 2021;25(4):209–212. 32654634 10.1089/omi.2020.0099

[56] Nie C, Trimpert J, Moon S, et al. In vitro efficacy of artemisia extracts against SARS-CoV-2. bioRxiv. 2021. 10.1186/s12985-021-01651-8 PMC8424155 34496903

[57] Chali BU, Melaku T, Berhanu N, et al. Traditional medicine practice in the context of COVID-19 pandemic: community claim in jimma zone, Oromia, Ethiopia. Infect Drug Resist. 2021;14:3773. 34557002 10.2147/IDR.S331434 PMC8453645

[58] Scherrer AM, Motti R, Weckerle CS. Traditional plant use in the areas of monte vesole and ascea, cilento national park (Campania, Southern Italy). J Ethnopharmacol. 2005;97(1):129–143. 15652287 10.1016/j.jep.2004.11.002

[59] Chaachouay N, Douira A, Zidane L. COVID-19, prevention and treatment with herbal medicine in the herbal markets of salé prefecture, North-Western Morocco. Eur J Integr Med. 2021;42:101285. 33520016 10.1016/j.eujim.2021.101285 PMC7836426

[60] Aanouz I, Belhassan A, El-Khatabi K, et al. Moroccan Medicinal plants as inhibitors against SARS-CoV-2 main protease: computational investigations. J Biomol Struct Dyn. 2021;39(8):2971–2979. DOI:10.1080/07391102.2020.1758790. 32306860 PMC7212546

[61] Mata R, Figueroa M, Navarrete A, et al. Chemistry and biology of selected Mexican medicinal plants. Prog Chem Org Nat Prod. 2019;108(2019):1–142. DOI:10.1007/978-3-030-01099-7_1. 30924013

[62] Rivero-Segura NA, Gomez-Verjan JC. In silico screening of natural products isolated from Mexican herbal medicines against COVID-19. Biomolecules. 2021;11(2):216. 33557097 10.3390/biom11020216 PMC7913859

[63] Kim D, Lee JY, Yang JS, et al. The architecture of SARS-CoV-2 transcriptome. Cell. 2020;181(4):914–921. DOI:10.1016/j.cell.2020.04.011. 32330414 PMC7179501

[64] Chan JF, Kok KH, Zhu Z, et al. Genomic characterization of the 2019 novel human-pathogenic coronavirus isolated from a patient with atypical pneumonia after visiting Wuhan. Emerg Microbes Infect. 2020;9(1):221–236. DOI:10.1080/22221751.2020.1719902. 31987001 PMC7067204

[65] Kim JM, Chung YS, Jo HJ, et al. Identification of Coronavirus isolated from a patient in Korea with COVID-19. Osong Public Health Res Perspect. 2020;11(1):3–7. DOI:10.24171/j.phrp.2020.11.1.02. 32149036 PMC7045880

[66] Zhou P, Yang XL, Wang XG, et al. A pneumonia outbreak associated with a new coronavirus of probable bat origin. Nature. 2020;579(7798):270–273. DOI:10.1038/s41586-020-2012-7. 32015507 PMC7095418

[67] Hoffmann M, Kleine-Weber H, Schroeder S, et al. SARS-CoV-2 cell entry depends on ACE2 and TMPRSS2 and is blocked by a clinically proven protease inhibitor. Cell. 2020;181(2):271–280. DOI:10.1016/j.cell.2020.02.052. 32142651 PMC7102627

[68] Poduri R, Joshi G, Jagadeesh G. Drugs targeting various stages of the SARS-CoV-2 life cycle: exploring promising drugs for the treatment of Covid-19. Cell Signal. 2020;74:109721. 32711111 10.1016/j.cellsig.2020.109721 PMC7375293

[69] Huang C, Wang Y, Li X, et al. Clinical features of patients infected with 2019 novel coronavirus in Wuhan, China. Lancet. 2020;395(10223):497–506. DOI:10.1016/S0140-6736(20)30183-5. 31986264 PMC7159299

[70] Fung TS, Liao Y, Liu DX. Regulation of stress responses and translational control by Coronavirus. Viruses. 2016;8(7):184. 10.3390/v8070184 PMC4974519 27384577

[71] Xiao H, Xu LH, Yamada Y, et al. Coronavirus spike protein inhibits host cell translation by interaction with eIF3f. Plos One. 2008;3(1):e1494. DOI:10.1371/journal.pone.0001494. 18231581 PMC2204050

[72] Lokugamage KG, Narayanan K, Nakagawa K, et al. Middle East respiratory syndrome Coronavirus nsp1 inhibits host gene expression by selectively targeting mrnas transcribed in the nucleus while sparing mRNAs of cytoplasmic origin. J Virol. 2015;89(21):10970–10981. DOI:10.1128/JVI.01352-15. 26311885 PMC4621111

[73] Lokugamage KG, Narayanan K, Huang C, et al. Severe acute respiratory syndrome coronavirus protein nsp1 is a novel eukaryotic translation inhibitor that represses multiple steps of translation initiation. J Virol. 2012;86(24):13598–13608. DOI:10.1128/JVI.01958-12. 23035226 PMC3503042

[74] Schubert K, Karousis ED, Jomaa A, et al. SARS-CoV-2 Nsp1 binds the ribosomal mRNA channel to inhibit translation. Nat Struct Mol Biol. 2020;27(10):959–966. DOI:10.1038/s41594-020-0511-8. 32908316

[75] Ma-Lauer Y, Carbajo-Lozoya J, Hein MY, et al. p53 down-regulates SARS coronavirus replication and is targeted by the SARS-unique domain and PLpro via E3 ubiquitin ligase RCHY1. Proc Natl Acad Sci U S A. 2016;113(35):E5192–E5201. DOI:10.1073/pnas.1603435113. 27519799 PMC5024628

[76] Wei J, Alfajaro MM, Hanna RE, et al. Genome-wide CRISPR screen reveals host genes that regulate SARS-CoV-2 infection. bioRxiv. 2020.

[77] Frieman M, Heise M, Baric R. SARS coronavirus and innate immunity. Virus Res. 2008;133(1):101–112. 17451827 10.1016/j.virusres.2007.03.015 PMC2292640

[78] Chen N, Zhou M, Dong X, et al. Epidemiological and clinical characteristics of 99 cases of 2019 novel coronavirus pneumonia in Wuhan, China: a descriptive study. Lancet. 2020;395(10223):507–513. DOI:10.1016/S0140-6736(20)30211-7. 32007143 PMC7135076

[79] Appelberg S, Gupta S, Svensson AS, et al. Dysregulation in Akt/mTOR/HIF-1 signaling identified by proteo-transcriptomics of SARS-CoV-2 infected cells. Emerg Microbes Infect. 2020;9(1):1748–1760. DOI:10.1080/22221751.2020.1799723. 32691695 PMC7473213

[80] Fung TS, Liu DX. Human Coronavirus: Host-Pathogen interaction. Annu Rev Microbiol. 2019;73(1):529–557. 31226023 10.1146/annurev-micro-020518-115759

[81] Min JS, Kim DE, Jin Y, et al. Kurarinone inhibits HCoV-OC43 infection by impairing the virus-induced autophagic flux in MRC-5 human lung cells. J Clin Med. 2020;9(7):2230. DOI:10.3390/jcm9072230. PMC7408680 32674356

[82] He T, Qu R, Qin C, et al. Potential mechanisms of Chinese herbal medicine that implicated in the treatment of COVID-19 related renal injury. Saudi Pharm J. 2020;28(9):1138–1148. DOI:10.1016/j.jsps.2020.08.002. 32837217 PMC7416081

[83] Shukla R, Singh S, Singh A, et al. Two pronged approach for prevention and therapy of COVID-19 (Sars-CoV-2) by a multi-targeted herbal drug, a component of ayurvedic decoction. Eur J Integr Med. 2021;43:101268. 33520014 10.1016/j.eujim.2020.101268 PMC7837292

[84] Roshdy WH, Rashed HA, Kandeil A, et al. EGYVIR: an immunomodulatory herbal extract with potent antiviral activity against SARS-CoV-2. Plos One. 2020;15(11):e241739. DOI:10.1371/journal.pone.0241739. PMC7673558 33206688

[85] Pan B, Fang S, Zhang J, et al. Chinese herbal compounds against SARS-CoV-2: puerarin and quercetin impair the binding of viral S-protein to ACE2 receptor. Comput Struct Biotec. 2020; 18: 3518–3527. 10.1016/j.csbj.2020.11.010 PMC7657012 33200026

[86] Siddiqui S, Upadhyay S, Ahmad R, et al. Virtual screening of phytoconstituents from miracle herb nigella sativa targeting nucleocapsid protein and papain-like protease of SARS-CoV-2 for COVID-19 treatment. J Biomol Struct Dyn 2020 1–21 10.1080/07391102.2020.1852117 PMC7738213 33289456

[87] Du A, Zheng R, Disoma C, et al. Epigallocatechin-3-gallate, an active ingredient of traditional Chinese medicines, inhibits the 3CLpro activity of SARS-CoV-2. Int J Biol Macromol. 2021;176:1–12. 33548314 10.1016/j.ijbiomac.2021.02.012 PMC7859723

[88] Lv Y, Wang S, Liang P, et al. Screening and evaluation of anti-SARS-CoV-2 components from Ephedra sinica by ACE2/CMC-HPLC-IT-TOF-MS approach. Anal Bioanal Chem. 2021;413(11):2995–3004. DOI:10.1007/s00216-021-03233-7. 33608752 PMC7895511

[89] Amparo TR, Seibert JB, Almeida TC, et al. In silico approach of secondary metabolites from Brazilian herbal medicines to search for potential drugs against SARS-CoV2. Phytother Res. 2021;35(8):4297–4308. DOI:10.1002/ptr.7097. 33797123 PMC8250981

[90] Chaturvedi M, Nagre K, Yadav JP. In silico approach for identification of natural compounds as potential COVID 19 main protease (Mpro) inhibitors. VirusDisease. 2021;1–5. DOI:10.1007/s13337-021-00701-7 PMC8214915 34179304

[91] Tejera E, Pérez-Castillo Y, Toscano G, et al. Computational modeling predicts potential effects of the herbal infusion “horchata” against COVID-19. Food Chem. 2022;366:130589. 34311241 10.1016/j.foodchem.2021.130589 PMC8314115

[92] Gurung AB, Ali MA, and Lee J, et al. Identification of SARS-CoV-2 inhibitors from extracts of Houttuynia cordata Thunb. Saudi J Biol Sci. 2021;28(12): 7517–7527. 34512097 10.1016/j.sjbs.2021.08.100 PMC8420092

[93] Cao R, Hu H, Li Y, et al. Anti-SARS-CoV-2 potential of artemisinins in vitro. Acs Infect Dis. 2020;6(9):2524–2531. DOI:10.1021/acsinfecdis.0c00522. 32786284

[94] Mu C, Sheng Y, Wang Q, et al. Potential compound from herbal food of rhizoma polygonati for treatment of COVID-19 analyzed by network pharmacology: viral and cancer signaling mechanisms. J Funct Foods. 2021;77:104149. 32837538 10.1016/j.jff.2020.104149 PMC7427583

[95] Yepes-Pérez AF, Herrera-Calderon O, Quintero-Saumeth J. Uncaria tomentosa (cat’s claw): a promising herbal medicine against SARS-CoV-2/ACE-2 junction and SARS-CoV-2 spike protein based on molecular modeling. J Biomol Struct Dyn. 2020;1–17. DOI:10.1080/07391102.2020.1837676 PMC7657399 33118480

[96] Huynh T, Wang H, Luan B. Structure-based lead optimization of herbal medicine rutin for inhibiting SARS-CoV-2ʹs main protease. Phys Chem Chem Phys. 2020;22(43):25335–25343. 33140777 10.1039/d0cp03867a

[97] Lu X, Yujing S, Jie SU, et al. Shufeng Jiedu, a promising herbal therapy for moderate COVID-19: antiviral and anti-inflammatory properties, pathways of bioactive compounds, and a clinical real-world pragmatic study. Phytomedicine. 2021;85:153390. 33158717 10.1016/j.phymed.2020.153390 PMC7581328

[98] Gupta S, Singh V, Varadwaj PK, et al. Secondary metabolites from spice and herbs as potential multitarget inhibitors of SARS-CoV-2 proteins. J Biomol Struct Dyn 2020 1–20 10.1080/07391102.2020.1837679 PMC7605658 33107812

[99] Balkrishna A, Haldar S, Singh H, et al. Coronil, a tri-herbal formulation, attenuates spike-protein-mediated SARS-CoV-2 viral entry into human alveolar epithelial cells and pro-inflammatory cytokines production by inhibiting spike protein-ACE-2 interaction. J Inflamm Res. 2021;14:869. 33758527 10.2147/JIR.S298242 PMC7981146

[100] Wen CC, Kuo YH, Jan JT, et al. Specific plant terpenoids and lignoids possess potent antiviral activities against severe acute respiratory syndrome coronavirus. J Med Chem. 2007;50(17):4087–4095. DOI:10.1021/jm070295s. 17663539

[101] Ryu YB, Park SJ, Kim YM, et al. SARS-CoV 3CLpro inhibitory effects of quinone-methide triterpenes from Tripterygium regelii. Bioorg Med Chem Lett. 2010;20(6):1873–1876. DOI:10.1016/j.bmcl.2010.01.152. 20167482 PMC7127101

[102] Park JY, Kim JH, Kim YM, et al. Tanshinones as selective and slow-binding inhibitors for SARS-CoV cysteine proteases. Bioorg Med Chem. 2012;20(19):5928–5935. DOI:10.1016/j.bmc.2012.07.038. 22884354 PMC7127169

[103] Park JY, Kim JH, Kwon JM, et al. Dieckol, a SARS-CoV 3CL(pro) inhibitor, isolated from the edible brown algae Ecklonia cava. Bioorg Med Chem. 2013;21(13):3730–3737. DOI:10.1016/j.bmc.2013.04.026. 23647823 PMC7126891

[104] Song YH, Kim DW, Curtis-Long MJ, et al. Papain-like protease (PLpro) inhibitory effects of cinnamic amides from Tribulus terrestris fruits. Biol Pharm Bull. 2014;37(6):1021–1028. DOI:10.1248/bpb.b14-00026. 24882413

[105] Park JY, Ko JA, Kim DW, et al. Chalcones isolated from Angelica keiskei inhibit cysteine proteases of SARS-CoV. J Enzyme Inhib Med Chem. 2016;31(1):23–30. DOI:10.3109/14756366.2014.1003215. 25683083

[106] Liu L-S *et al* The Effects and Mechanism of Yinqiao Powder on Upper Respiratory Tract Infection . International Journal of Biotechnology for Wellness Industries. 2(4). 57–60

[107] Ding Y, Zeng L, Li R, et al. The Chinese prescription lianhuaqingwen capsule exerts anti-influenza activity through the inhibition of viral propagation and impacts immune function. BMC Complement Altern Med. 2017;17(1):130. DOI:10.1186/s12906-017-1585-7. 28235408 PMC5324200

[108] Gao J, Li J, Shao X, et al. Antiinflammatory and immunoregulatory effects of total glucosides of Yupingfeng powder. Chin Med J (Engl). 2009;122(14):1636–1641. 19719964

[109] Su H, Yao S, Zhao W, et al. Anti-SARS-CoV-2 activities in vitro of Shuanghuanglian preparations and bioactive ingredients. Acta Pharmacol Sin. 2020;41(9):1167–1177. DOI:10.1038/s41401-020-0483-6. 32737471 PMC7393338

[110] Zeng M, Li L, Wu Z. Traditional Chinese medicine Lianhua Qingwen treating Corona virus disease 2019 (COVID-19): meta-analysis of randomized controlled trials. Plos One. 2020;15(9):e238828. 10.1371/journal.pone.0238828 PMC7485773 32915877

[111] Cheng Dezhong LY. Clinical effectiveness and case analysis in 54 NCP patients treated with Lianhuaqingwen Granules. World Chin Med. 2020;15(15):150–154.

[112] Lv R *et al*. Clinical observation on 63 cases of suspected cases of new Coronavirus pneumonia treated by Chinese medicine lianhua qingwen. J Tradit Chin Med. 2020.

[113] Yao K *et al*. Retrospective clinical analysis on treatment of novel coronavirus-infected pneumonia with traditional Chinese medicine lianhua qingwen. Chin J Exp Tradit Med Formul. 2020;

[114] Li H, Yang L, Liu FF, et al. Overview of therapeutic drug research for COVID-19 in China. Acta Pharmacol Sin. 2020;41(9):1133–1140. DOI:10.1038/s41401-020-0438-y. 32555446 PMC7298161

[115] Li T, Lu H, Zhang W. Clinical observation and management of COVID-19 patients. Emerg Microbes Infect. 2020;9(1):687–690. 32208840 10.1080/22221751.2020.1741327 PMC7103696

[116] Hu K, Guan WJ, Bi Y, et al. Efficacy and safety of Lianhuaqingwen capsules, a repurposed Chinese herb, in patients with coronavirus disease 2019: a multicenter, prospective, randomized controlled trial. Phytomedicine. 2020;85:153242. 33867046 10.1016/j.phymed.2020.153242 PMC7229744

[117] Runfeng L, Yunlong H, Jicheng H, et al. Lianhuaqingwen exerts anti-viral and anti-inflammatory activity against novel coronavirus (SARS-CoV-2). Pharmacol Res. 2020;156:104761. 32205232 10.1016/j.phrs.2020.104761 PMC7102548

[118] Chinese traditional and herbal drugs. 2020.

[119] Liu J, Jiang Y, Liu Y, et al. Yindan Jiedu granules, a traditional Chinese medicinal formulation, as a potential treatment for Coronavirus disease 2019. Front Pharmacol. 2021;11:2449. 10.3389/fphar.2020.634266 PMC7957926 33732148

[120] Feng J, Fang B, and Zhou D, et al. Clinical effect of traditional Chinese Medicine Shenhuang Granule in Critically Ill Patients with COVID-19: a Single-Centered, Retrospective, Observational Study. J Microbiol Biotechnol. 2021 31 3 380–386 ; 33746189 10.4014/jmb.2009.09029 PMC9705840

[121] Hetrick B, Yu D, Olanrewaju AA, et al. A traditional medicine, respiratory detox shot (RDS), inhibits the infection of SARS-CoV, SARS-CoV-2, and the influenza A virus in vitro. Cell Biosci. 2021;11(1):1–12. 34051873 10.1186/s13578-021-00609-1 PMC8164078

[122] Wang Y, Liu Y, Lv Q, et al. Effect and safety of Chinese herbal medicine granules in patients with severe coronavirus disease 2019 in Wuhan, China: a retrospective, single-center study with propensity score matching. Phytomedicine. 2020;85:153404. 33637412 10.1016/j.phymed.2020.153404 PMC7642753

[123] Shi N, Guo L, Liu B, et al. Efficacy and safety of Chinese herbal medicine versus lopinavir-ritonavir in adult patients with coronavirus disease 2019: a non-randomized controlled trial. Phytomedicine. 2021;81:153367. 33260064 10.1016/j.phymed.2020.153367 PMC7543765

[124] Liang S, Zhang Y, Shen C, et al. Chinese herbal medicine used with or without conventional Western therapy for COVID-19: an evidence review of clinical studies. Front Pharmacol. 2021;11:2321. 10.3389/fphar.2020.583450 PMC7953048 33716720

[125] Liu L, Shi F, and Tu P, et al. Arbidol combined with the Chinese medicine Lianhuaqingwen capsule versus arbidol alone in the treatment of COVID-19. Medicine (Baltimore). 2021;100(4):e24475. 33530261 10.1097/MD.0000000000024475 PMC7850685

[126] Jin W, Qiao L, Li Y. Effects of shen-fu injection on lipopolysaccharide-induced NF-κB activity and cytokine release in rat alveolar macrophages. Acta Medicinae Universitatis Scientiae Et Technologiae Huazhong. 2009.

[127] Xiu-Juan C, Shuai Z, and Yi-Ping J, et al. Mechanism of reduning injection on anti-acute lung injury in rats based on cytokine storm. Acta Pharmaceutica Sinica. 2015;46(2): 236–239

[128] Han L, Zhang Y, and Li H, et al. Network pharmacologic molecular mechanism of shenmai injection in treatment of COVID-19 combined with coronary heart disease. Acta Pharmaceutica Sinica. 2020;51(9): 2334–2344

[129] Ma Q, Xie Y, Wang Z, et al. Efficacy and safety of reDuNing injection as a treatment for COVID-19 and its inhibitory effect against SARS-CoV-2. J Ethnopharmacol. 2021;279:114367. 34174375 10.1016/j.jep.2021.114367 PMC8223030

[130] Zhang XY, Lv L, Zhou YL, et al. Efficacy and safety of Xiyanping injection in the treatment of COVID-19: a multicenter, prospective, open-label and randomized controlled trial. Phytother Res. 2021;35(8):4401–4410. DOI:10.1002/ptr.7141. 33979464 PMC8242486

[131] de Wilde AH, Snijder EJ, Kikkert M, et al. Host factors in Coronavirus replication. Curr Top Microbiol Immunol. 2018;419:1–42. 28643204 10.1007/82_2017_25 PMC7119980

[132] Fehr AR, Perlman S. Coronaviruses: an overview of their replication and pathogenesis. Methods Mol Biol. 2015;1282:1–23. 25720466 10.1007/978-1-4939-2438-7_1 PMC4369385

[133] Báez-Santos YM, St JS, Mesecar AD. The SARS-coronavirus papain-like protease: structure, function and inhibition by designed antiviral compounds. Antiviral Res. 2015;115:21–38. 25554382 10.1016/j.antiviral.2014.12.015 PMC5896749

[134] Morse JS, Lalonde T, Xu S, et al. Learning from the past: possible urgent prevention and treatment options for severe acute respiratory infections caused by 2019-nCoV. Chembiochem. 2020;21(5):730–738. DOI:10.1002/cbic.202000047. 32022370 PMC7162020

[135] Wu, H *et al* Preliminary exploration of the mechanism of Qingfei Paidu decoction against novel coronavirus pneumonia based on network pharmacology. Acta Pharmaceutica Sinica. 2020. 51 3:2317–2325

[136] Meng XY, Zhang HX, Mezei M, et al. Molecular docking: a powerful approach for structure-based drug discovery. Curr Comput Aided Drug Des. 2011;7(2):146–157. DOI:10.2174/157340911795677602. 21534921 PMC3151162

[137] Nguyen TT, Woo HJ, Kang HK, et al. Flavonoid-mediated inhibition of SARS coronavirus 3C-like protease expressed in Pichia pastoris. Biotechnol Lett. 2012;34(5):831–838. DOI:10.1007/s10529-011-0845-8. 22350287 PMC7087583

[138] Enmozhi SK, Raja K, Sebastine I, et al. Andrographolide as a potential inhibitor of SARS-CoV-2 main protease: an in silico approach. J Biomol Struct Dyn 2020 1–7 10.1080/07391102.2020.1760136 PMC7212536 32329419

[139] Cinatl J, Morgenstern B, Bauer G, et al. Glycyrrhizin, an active component of liquorice roots, and replication of SARS-associated coronavirus. Lancet. 2003;361(9374):2045–2046. DOI:10.1016/S0140-6736(03)13615-X. 12814717 PMC7112442

[140] Su H, Yao S, Zhao W, et al. Discovery of baicalin and baicalein as novel, natural product inhibitors of SARS-CoV-2 3CL protease in vitro. bioRxiv. 2020;2020–2024.

[141] Yu Y, Zhang Y, Wang S, et al. Inhibition effects of patchouli alcohol against influenza a virus through targeting cellular PI3K/Akt and ERK/MAPK signaling pathways. Virol J. 2019;16(1):163. DOI:10.1186/s12985-019-1266-x. 31870450 PMC6929483

[142] Ryu YB, Jeong HJ, Kim JH, et al. Biflavonoids from Torreya nucifera displaying SARS-CoV 3CL(pro) inhibition. Bioorg Med Chem. 2010;18(22):7940–7947. DOI:10.1016/j.bmc.2010.09.035. 20934345 PMC7126309

[143] Yu S, Wang J, Shen H. Network pharmacology-based analysis of the role of traditional Chinese herbal medicines in the treatment of COVID-19. Ann Palliat Med. 2020;9(2):437–446. 32233641 10.21037/apm.2020.03.27

[144] Ling X, Tao J, and Sun X, et al. Exploring material basis and mechanism of lianhua qingwen prescription against coronavirus based on network pharmacology. Acta Pharmaceutica Sinica. 2020;51(7): 1723–1730

[145] Chen, H , Du, Q Potential natural compounds for preventing SARS-CoV-2 (2019-nCoV) infection. Preprints. 2020.

[146] Wu, H *et al* Preliminary exploration of the mechanism of Qingfei Paidu decoction against novel coronavirus pneumonia based on network pharmacology and molecular docking technolog. Acta Pharmaceutica Sinica. 2020;55(3):374–383.

[147] Tao Q, Du J, Li X, et al. Network pharmacology and molecular docking analysis on molecular targets and mechanisms of Huashi Baidu formula in the treatment of COVID-19. Drug Dev Ind Pharm. 2020;46(8):1345–1353. DOI:10.1080/03639045.2020.1788070. 32643448 PMC7441778

[148] Jimilihan SMY. Study on the active components in the adjuvant treatment of novel coronavirus pneumonia (COVID-19) with jinhua qinggan granules based on network pharmacology and molecular docking. Journal of Chinese Medicinal Materials. 2020;3:1–10.

[149] Fu FZWY S. Based on network pharmacology and high-throughput molecular docking to study the potential molecular mechanism of active compounds tthat bind SARS-Cov-2 specific target protein in Jinhua Qinggan granules to interfere with CovID-19. Modernization of Traditional Chinese Medicine and Materia Materia-World Science and Technology. 2020;1:1–10.

[150] Shahrajabian MH, Sun W, Soleymani A, et al. Traditional herbal medicines to overcome stress, anxiety and improve mental health in outbreaks of human coronaviruses. Phytother Res. 2021;35(3):1237–1247. DOI:10.1002/ptr.6888. 33350538

[151] Pan F, Ye T, Sun P, et al. Time course of lung changes at chest CT during recovery from Coronavirus disease 2019 (COVID-19). Radiology. 2020;295(3):715–721. DOI:10.1148/radiol.2020200370. 32053470 PMC7233367

[152] Hadjadj J, Yatim N, Barnabei L, et al. Impaired type I interferon activity and inflammatory responses in severe COVID-19 patients. Science. 2020;369(6504):718–724. DOI:10.1126/science.abc6027. 32661059 PMC7402632

[153] Yang G, Tan Z, Zhou L, et al. Effects of Angiotensin II receptor blockers and ACE (Angiotensin-Converting Enzyme) inhibitors on virus infection, inflammatory status, and clinical outcomes in patients with COVID-19 and hypertension: a single-center retrospective study. Hypertension. 2020;76(1):51–58. DOI:10.1161/HYPERTENSIONAHA.120.15143. 32348166

[154] Mehta P, McAuley DF, Brown M, et al. COVID-19: consider cytokine storm syndromes and immunosuppression. Lancet. 2020;395(10229):1033–1034. DOI:10.1016/S0140-6736(20)30628-0. 32192578 PMC7270045

[155] Zhang DH, Wu KL, Zhang X, et al. In silico screening of Chinese herbal medicines with the potential to directly inhibit 2019 novel coronavirus. J Integr Med. 2020;18(2):152–158. DOI:10.1016/j.joim.2020.02.005. 32113846 PMC7102521

[156] Li L, Zhang Z, Zhou W, et al. Lianhua Qingwen prescription for Coronavirus disease 2019 (COVID-19) treatment: advances and prospects. Biomed Pharmacother. 2020;130:110641. 34321172 10.1016/j.biopha.2020.110641 PMC7437484

[157] Zhao J, Tian S, and Yang J, et al. Investigating mechanism of Qing-Fei-Pai-Du-Tang for treatment of COVID-19 by network pharmacology. Chin. Trad. Herbal Drugs. 2020;4(51):829–835.

[158] Chen J, Wang Y, Gao Y, et al. Protection against COVID-19 injury by qingfei paidu decoction via anti-viral, anti-inflammatory activity and metabolic programming. Biomed Pharmacother. 2020;129:110281. 32554251 10.1016/j.biopha.2020.110281 PMC7247521

[159] Huang YF, Bai C, He F, et al. Review on the potential action mechanisms of Chinese medicines in treating Coronavirus disease 2019 (COVID-19). Pharmacol Res. 2020;158:104939. 32445956 10.1016/j.phrs.2020.104939 PMC7239792

[160] Liu Y, Wei P, and Qiu Z. Study on mechanism of Shufeng Jiedu granules in treating novel coronavirus pneumonia based on network pharmacology. 2019 International Conference on Biotechnology and Bioengineering(9th ICBB) . *2020-01-01* 2020: AIP Publishing LLC; 2020. 20013

[161] Ren Y, Yao MC, Huo XQ, et al. Study on treatment of “cytokine storm” by anti-2019-nCoV prescriptions based on arachidonic acid metabolic pathway. Zhongguo Zhong Yao Za Zhi. 2020;45(6):1225–1231. DOI:10.19540/j.cnki.cjcmm.20200224.405. 32281329

[162] Cao C, Cui Y, Chu Y, et al. Investigation on mechanism and active components of shufeng jiedu capsule in treatment of COVID-19 based on network pharmacology and molecular docking. Chin. Trad. Herbal Drugs. 2020;51(9): 2283–2296

[163] Shen FFZY. The potential targets and mechanisms of shufeng jiedu capsule for novel coronavirus pneumonia (COVID-19) based on network pharmacology and molecular docking. Guiding J Tradit Chin Med Pharm. 2020;5(26):8–15.

[164] Zhang T. Basic study on treatment of COVID-19 with shufeng jiedu capsule and research and development ideas of new Chinese materia medica against COVID-19. Chin. Trad. Herbal Drugs. 2020;9(51):2273–2282.

[165] Yang R, Liu H, Bai C, et al. Chemical composition and pharmacological mechanism of Qingfei Paidu decoction and Ma Xing Shi Gan decoction against Coronavirus disease 2019 (COVID-19): in silico and experimental study. Pharmacol Res. 2020;157:104820. 32360484 10.1016/j.phrs.2020.104820 PMC7194979

[166] Ma Q, Pan W, Li R, et al. Liu Shen capsule shows antiviral and anti-inflammatory abilities against novel coronavirus SARS-CoV-2 via suppression of NF-κB signaling pathway. Pharmacol Res. 2020;158:104850. 32360580 10.1016/j.phrs.2020.104850 PMC7192119

[167] Xiong W, Wang G, Du J, et al. Efficacy of herbal medicine (Xuanfei Baidu decoction) combined with conventional drug in treating COVID-19: a pilot randomized clinical trial. Integr Med Res. 2020;9(3):100489. DOI:10.1016/j.imr.2020.100489. 32874913 PMC7452296

[168] Zhang X, Xue Y, Chen X, et al. Effects of tanreqing capsule on the negative conversion time of nucleic acid in patients with COVID-19: a retrospective cohort study. J Integr Med. 2021;19(1):36–41. DOI:10.1016/j.joim.2020.10.002. 33069626 PMC7532751

[169] Cui Y, Xin H, Tao Y, et al. Arenaria kansuensis attenuates pulmonary fibrosis in mice via the activation of Nrf2 pathway and the inhibition of NF‐kB/TGF‐beta1/Smad2/3 pathway. Phytother Res. 2021;35(2):974–986. DOI:10.1002/ptr.6857. 32996197

[170] De Jin XA, Zhang Y, and Zhao S, et al. Potential mechanism prediction of herbal medicine for pulmonary fibrosis associated with SARS-CoV-2 infection based on network analysis and molecular docking. Front Pharmacol. 2021;12:602218. doi:10.3389/fphar.2021.602218. 33986661 PMC8112227

[171] Yang Y. Use of herbal drugs to treat COVID-19 should be with caution. Lancet. 2020;395(10238):1689–1690. 32422123 10.1016/S0140-6736(20)31143-0 PMC7228688

